# Supplementary material for: Annelid functional genomics reveal the origins of bilaterian life cycles
Source: Nature. 2023 Jan 25;615(7950):105–10. doi: 10.1038/s41586-022-05636-7 (PMC9977687; doi:10.1038/s41586-022-05636-7)
Supplement: Supplementary file 1 — This file contains 33 Supplementary Figs. and legends for 94 Supplementary Tables. [file 41586_2022_5636_MOESM1_ESM.pdf]

nature portfolio

https://doi.org/10.1038/s41586-022-05636-7

Supplementary information

Annelid functional genomics reveal the origins of bilaterian life cycles

In the format provided by the
authors and unedited

Supplementary Information

Annelid functional genomics reveal the origins of bilaterian life cycles

Francisco M. Martín-Zamora[1,§], Yan Liang[1,§], Kero Guynes[1], Allan M. Carrillo-Baltodano[1], Billie E. Davies[1], Rory D. Donnellan[1], Yongkai Tan[2], Giacomo Moggioli[1], Océane Seudre[1], Martin Tran[1,3], Kate Mortimer[4], Nicholas M. Luscombe[2], Andreas Hejnol[5,6], Ferdinand Marlétaz[7,*], José M. Martín-Durán[1,*]

[1]School of Biological and Behavioural Sciences, Queen Mary University of London, London, United Kingdom
[2]Genomics and Regulatory Systems Unit, Okinawa Institute of Science and Technology Graduate University, Okinawa, Japan
[3]Department of Infectious Disease, Imperial College London, London, United Kingdom
[4]Department of Natural Sciences, Amgueddfa Cymru – Museum Wales, Cardiff, United Kingdom
[5]Department of Biological Sciences, University of Bergen, Bergen, Norway
[6]Institute of Zoology and Evolutionary Research, Faculty of Biological Sciences, Friedrich Schiller University Jena, Jena, Germany
[7]Department of Genetics, Evolution and Environment, University College London, London, United Kingdom

§ These authors contributed equally.
* Correspondence: José M. Martín-Durán (chema.martin@qmul.ac.uk), Ferdinand Marlétaz (f.marletaz@ucl.ac.uk)

This PDF file includes:

- 33 Supplementary Figures
- 94 Supplementary Tables legends

Index of Supplementary Figures:

- **Supplementary Figure 1.** *O. fusiformis* genome sequencing, assembly, and annotation.
- **Supplementary Figure 2.** Quality control and sample correlation of stage-specific RNA-seq samples of *O. fusiformis* and *C. teleta*.
- **Supplementary Figure 3.** Transcription factor repertoire profiling.
- **Supplementary Figure 4.** GO terms enrichment of RNA-seq clusters of *O. fusiformis*.
- **Supplementary Figure 5.** GO terms enrichment of RNA-seq clusters of *C. teleta.*
- **Supplementary Figure 6.** GO terms enrichment of RNA-seq clusters of *D. gyrociliatus*.
- **Supplementary Figure 7.** Benchmarking of clustering of GO terms enriched in annelid RNA-seq clusters.
- **Supplementary Figure 8.** Clustering of GO terms enriched in annelid RNA-seq clusters.

- **Supplementary Figure 8.** Clustering of GO terms enriched in annelid RNA-seq clusters.

- **Supplementary Figure 9.** Weighted gene co-expression network analyses.
- **Supplementary Figure 10.** GO terms enrichment of WGCNA modules of *O. fusiformis*.
- **Supplementary Figure 11.** GO terms enrichment of WGCNA modules of *C. teleta*.
- **Supplementary Figure 12.** Expression dynamics of transcription factors consistently under heterochronic shifts between indirect and direct development.
- **Supplementary Figure 13.** GO terms enrichment of gene sets with different timings of expression between *O. fusiformis* and *C. teleta.*
- **Supplementary Figure 14.** Expression dynamics of transcription factors under heterochronic shifts between larval types.
- **Supplementary Figure 15.** Heterochronic shift of the annelid chitin synthesis pathway genes between larval types.
- **Supplementary Figure 16.** Heterochronic shift of the autophagy pathway genes between larval types.
- **Supplementary Figure 17.** Quality control and sample correlation of stage-specific ATAC-seq samples of *O. fusiformis* and *C. teleta.*
- **Supplementary Figure 18.** Accessible chromatin landscape profiling.
- **Supplementary Figure 19.** GO terms enrichment of gene sets regulated by ATAC-seq peak clusters in *O. fusiformis*.
- **Supplementary Figure 20.** GO terms enrichment of gene sets regulated by ATAC-seq peak clusters in *C. teleta*.
- **Supplementary Figure 21.** Benchmarking of clustering of GO terms enriched in gene sets regulated by annelid ATAC-seq peak clusters.

- **Supplementary Figure 22.** Clustering of GO terms enriched in gene sets regulated by annelid ATAC-seq peak clusters.
- **Supplementary Figure 23.** Annelid time-course of biological processes regulated by accessible chromatin
- **Supplementary Figure 24.** Cross-database known motif analysis benchmarking.
- **Supplementary Figure 25.** Accessibility dynamics of the full motif archetype set.
- **Supplementary Figure 26.** Transcription factor footprinting of the full motif archetype set.
- **Supplementary Figure 27.** Accessibility dynamics of the common annelid motif archetype set.
- **Supplementary Figure 28.** Transcription factor footprinting of the common annelid motif archetype set.
- **Supplementary Figure 29.** Cross-species comparison of transcription factor binding dynamics in the common annelid motif archetype set.
- **Supplementary Figure 30.** Differential transcription factor binding.
- **Supplementary Figure 31.** Transcription factor binding dynamics in peaks of the *Hox* genes cluster.
- **Supplementary Figure 32.** Developmental expression dynamics of phylum- and species-specific genes across Metazoa.
- **Supplementary Figure 33.** *Hox* genes developmental expression dynamics across Metazoa.

Index of Supplementary Tables:

- **Supplementary Table 1.** Repetitive elements genome composition in annelids.
- **Supplementary Table 2.** Genomes and annotation files used for gene family evolution analyses.
- **Supplementary Table 3.** Gene family evolution analysis in 22 metazoan lineages.
- **Supplementary Table 4.** Statistics of gene family evolution analysis.
- **Supplementary Table 5.** Gene family gains in *O. fusiformis*. *P*-values were derived from upper-tail hypergeometric tests.
- **Supplementary Table 6.** Gene family losses in *O. fusiformis*.
- **Supplementary Table 7.** Analysis of pre- and metazoan orthogroups retention.
- **Supplementary Table 8.** Datasets used for annelid *chordin* mining.
- **Supplementary Table 9.** Summary of mutual best hit (MBH) analysis and translated peptides for all 104 unique *chordin* annelid candidates.
- **Supplementary Table 10.** Curated sequences used for *chordin* orthology assignment.
- **Supplementary Table 11.** List of *chordin* annelid candidates, presence or absence in phylogenetic analyses A and B, and associated exclusion criteria.
- **Supplementary Table 12.** Summary of chordin presence/absence across annelids.
- **Supplementary Table 13.** Statistics of RNA-seq libraries for *O. fusiformis*.
- **Supplementary Table 14.** Stage-specific TPM gene expression matrix for *O. fusiformis*.
- **Supplementary Table 15.** Stage-specific DESeq2 gene expression matrix for *O. fusiformis*.
- **Supplementary Table 16.** Statistics of RNA-seq libraries for *C. teleta*.
- **Supplementary Table 17.** Stage-specific TPM gene expression matrix for *C. teleta*.
- **Supplementary Table 18.** Stage-specific DESeq2 gene expression matrix for *C. teleta*.
- **Supplementary Table 19.** Statistics of RNA-seq libraries for *D. gyrociliatus*.
- **Supplementary Table 20.** Stage-specific TPM gene expression matrix for *D. gyrociliatus*.
- **Supplementary Table 21.** Stage-specific DESeq2 gene expression matrix for *D. gyrociliatus*.
- **Supplementary Table 22.** Stages of minimal transcriptome divergence to each *O. fusiformis* or *C. teleta* stage, calculated from the full single copy ortholog set.
- **Supplementary Table 23.** Genome annotation based on RNA-seq clusters and WGCNA modules for *O. fusiformis*.
- **Supplementary Table 24.** Genome annotation based on RNA-seq clusters and WGCNA modules for *C. teleta*.
- **Supplementary Table 25.** Genome annotation based on RNA-seq clusters for *D. gyrociliatus*.
- **Supplementary Table 26.** Statistics of RNA-seq clustering for all 3 species.

- **Supplementary Table 27.** Statistics of gene ontology terms enrichment of RNA-seq clusters for *O. fusiformis*. Top 30 significant GO terms for each cluster are shown.
- **Supplementary Table 28.** Statistics of gene ontology terms enrichment of RNA-seq clusters for *C. teleta*. Top 30 significant GO terms for each cluster are shown.
- **Supplementary Table 29.** Statistics of gene ontology terms enrichment of RNA-seq clusters for *D. gyrociliatus*. Top 30 significant GO terms for each cluster are shown.
- **Supplementary Table 30.** Statistics of gene ontology terms enrichment of WGCNA modules for *O. fusiformis*. Top 15 significant GO terms for each module are shown.
- **Supplementary Table 31.** Statistics of gene ontology terms enrichment of WGCNA modules for *C. teleta*. Top 15 significant GO terms for each module are shown.
- **Supplementary Table 32**. Transcription factor PFAM domains used for transcription factor mining and classification.
- **Supplementary Table 33**. Transcription factor repertoire in all 3 species.
- **Supplementary Table 34**. List of common genes from *O. fusiformis* and *C. teleta* late clusters shifted to *D. gyrociliatus* early clusters.
- **Supplementary Table 35**. List of common transcription factors from *O. fusiformis* and *C. teleta* late clusters shifted to *D. gyrociliatus* early clusters.
- **Supplementary Table 36**. List of genes from *O. fusiformis* late clusters shifted to *C. teleta* early clusters.
- **Supplementary Table 37**. List of transcription factors from *O. fusiformis* late clusters shifted to *C. teleta* early clusters.
- **Supplementary Table 38**. List of genes from *O. fusiformis* early clusters shifted to *C. teleta* late clusters.
- **Supplementary Table 39**. List of transcription factors from *O. fusiformis* early clusters shifted to *C. teleta* late clusters.
- **Supplementary Table 40**. Statistics of gene ontology terms enrichment of gene sets shifted between early and late clusters for *O. fusiformis* and *C. teleta*. Top 30 significant GO terms for each gene set are shown.
- **Supplementary Table 41**. KEGG BlastKOALA annotation of genes from *O. fusiformis* late clusters shifted to *C. teleta* early clusters.
- **Supplementary Table 42.** KEGG BlastKOALA annotation of genes from *O. fusiformis* early clusters shifted to *C. teleta* late clusters.
- **Supplementary Table 43.** Enzymatic activities and associated genes involved in chitin synthesis pathway in insects, fungi, and annelids.
- **Supplementary Table 44.** Expression deployment timing of genes involved in chitin synthesis in *O. fusiformis* and *C. teleta*.
- **Supplementary Table 45.** KEGG animal/human autophagy pathway genes used for gene mining and pathway completeness analysis.
- **Supplementary Table 46.** Common single-copy *O. fusiformis* and *C. teleta* orthologs to animal/human autophagy pathway genes, and their expression deployment timing.
- **Supplementary Table 47.** Curated sequences used for *Hox* genes orthology assignment.

- **Supplementary Table 48.** Datasets used for *Hox* gene expression analysis during *Urechis unicinctus* development.
- **Supplementary Table 49.** Statistics of adult tissues RNA-seq libraries for *O. fusiformis*.
- **Supplementary Table 50.** Stage-specific TPM gene expression matrix for adult tissue samples of *O. fusiformis*.
- **Supplementary Table 51.** Stage-specific DESeq2 gene expression matrix for adult tissue samples of *O. fusiformis*.
- **Supplementary Table 52**. List of anterior genes expressed in both the adult head tissue and the adult head plus the two anterior most segments tissue in *O. fusiformis*.
- **Supplementary Table 53**. List of posterior genes expressed in both the adult body wall tissue and the adult tail in *O. fusiformis*.
- **Supplementary Table 54**. DESeq2 expression matrix of *in situ* hybridisation-validated anterior, trunk, and posterior markers of *O. fusiformis*.
- **Supplementary Table 55**. DESeq2 expression matrix of *in situ* hybridisation-validated anterior, trunk, and posterior markers of *C. teleta.*
- **Supplementary Table 56**. DESeq2 expression matrix of *in situ* hybridisation-validated anterior, and posterior markers of *D. gyrociliatus*.
- **Supplementary Table 57.** Primers used for ATAC-seq library preparation for *O. fusiformis*.
- **Supplementary Table 58.** Primers used for ATAC-seq library preparation for *C. teleta*.
- **Supplementary Table 59.** Statistics of ATAC-seq experiments of *O. fusiformis*.
- **Supplementary Table 60.** Statistics of ATAC-seq experiments of *C. teleta*.
- **Supplementary Table 61.** Genomic location and normalised stage-wise accessibility of the consensus ATAC-seq peak set of *O. fusiformis*.
- **Supplementary Table 62.** Normalised stage-wise occupancy of the consensus ATAC-seq peak set of *O. fusiformis*.
- **Supplementary Table 63.** Annotation by genomic feature and correlation with expression of nearest gene of the consensus ATAC-seq peak set of *O. fusiformis*.
- **Supplementary Table 64.** Genomic location and normalised stage-wise accessibility of the consensus ATAC-seq peak set of *O. fusiformis*.
- **Supplementary Table 65.** Normalised stage-wise occupancy of the consensus ATAC-seq peak set of *O. fusiformis*.
- **Supplementary Table 66.** Annotation by genomic feature and correlation with expression of nearest gene of the consensus ATAC-seq peak set of *O. fusiformis*.
- **Supplementary Table 67.** Cluster annotation of the consensus ATAC-seq peak set of *O. fusiformis*.
- **Supplementary Table 68.** Cluster annotation of the consensus ATAC-seq peak set of *O. fusiformis*.
- **Supplementary Table 69.** Statistics of ATAC-seq peak clustering for *O. fusiformis* and *C. teleta*.

- **Supplementary Table 70.** Statistics of gene ontology terms enrichment of gene sets regulated by ATAC-seq peak clusters for *O. fusiformis*. Top 30 significant GO terms for each cluster are shown.
- **Supplementary Table 71.** Statistics of gene ontology terms enrichment of gene sets regulated by ATAC-seq peak clusters for *C. teleta*. Top 30 significant GO terms for each cluster are shown.
- **Supplementary Table 72.** Motif discovery and enrichment analysis in *O. fusiformis*.
- **Supplementary Table 73.** Motif discovery and enrichment analysis in *C. teleta*.
- **Supplementary Table 74.** Cross-database known motif analysis benchmarking and manual curation of clustered motif archetypes.
- **Supplementary Table 75.** Motif archetype counts in the consensus ATAC-seq peak of *O. fusiformis*.
- **Supplementary Table 76.** Motif archetype counts in the consensus ATAC-seq peak of *C. teleta*.
- **Supplementary Table 77.** Normalised stage-wise accessibility of the motif archetypes in *O. fusiformis*.
- **Supplementary Table 78.** Normalised stage-wise accessibility of the motif archetypes in *C. teleta*.
- **Supplementary Table 79.** Transcription factor footprinting of the motif archetypes in *O. fusiformis*. Total and stage-wise binding score, and differential binding analysis from pair-wise comparisons between consecutive developmental stages are shown.
- **Supplementary Table 80.** Transcription factor footprinting of the motif archetypes in *C. teleta*. Total and stage-wise binding score, and differential binding analysis from pair-wise comparisons between consecutive developmental stages are shown.
- **Supplementary Table 81.** Total and peak-wise motif count in peaks from the consensus ATAC-seq peak set annotated to genes in the *Hox* cluster for *O. fusiformis*.
- **Supplementary Table 82.** Total and peak-wise motif count in peaks from the consensus ATAC-seq peak set annotated to genes in the *Hox* cluster for *C. teleta*.
- **Supplementary Table 83.** List of genes with an annotated peak with an annelid HOX/CDX/EVX bound motif at the competent larva stage of *O. fusiformis*.
- **Supplementary Table 84.** List of genes with an annotated peak with an annelid HOX/CDX/EVX bound motif at the stage 4tt larva stage of *C. teleta*.
- **Supplementary Table 85.** Phylostratigraphic genome annotation of *O. fusiformis*.
- **Supplementary Table 86.** Phylostratigraphic genome annotation of *C. teleta*.
- **Supplementary Table 87.** Phylostratigraphic genome annotation of *D. gyrociliatus*.
- **Supplementary Table 88.** Developmental RNA-seq time course datasets used for metazoan comparative transcriptomics analyses.
- **Supplementary Table 89.** Single copy ortholog number for all one-to-one comparisons between all 11 metazoan lineages.
- **Supplementary Table 90.** Number of transcription factor orthologs and their contribution to the full single copy ortholog set between *O. fusiformis* and the remaining 10 metazoan lineages.

- **Supplementary Table 91.** Statistics of gene ontology terms enrichment of gene sets with very low gene-wise Jensen-Shannon divergence between the mitraria larva of *O. fusiformis* and the stages of minimal transcriptomic divergence of *C. teleta*, *C. gigas*, *S. purpuratus*, and *N. vectensis*. Top 30 significant GO terms for each gene set are shown.
- **Supplementary Table 92.** List of transcription factors with very low gene-wise Jensen-Shannon divergence between the mitraria larva of *O. fusiformis* and stages of minimal transcriptomic divergence of *C. teleta*, *C. gigas*, *S. purpuratus*, and *N. vectensis*.
- **Supplementary Table 93.** Literature review on *Hox* genes deployment timing across bilaterian lineages.
- **Supplementary Table 94.** Gene identifiers of *Hox* genes used for stage-wise *Hox* gene expression profiling across all 12 studied metazoan lineages.

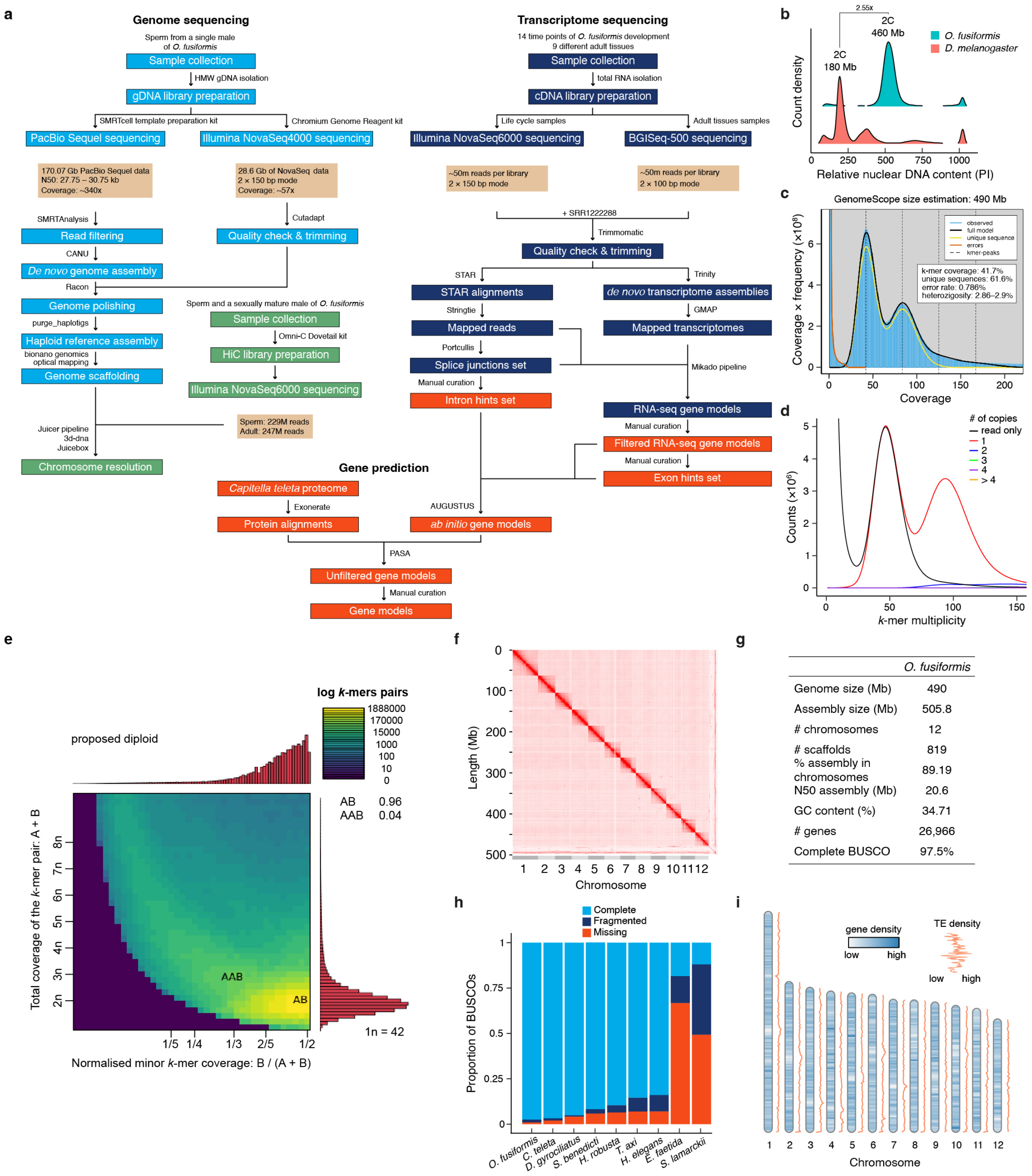

Supplementary Figure 1 | *O. fusiformis* genome sequencing, assembly, and annotation. **a,** Flow charts summarising genome (light blue) and transcriptome sequencing (dark blue), chromosome-level scaffolding (green) and gene prediction (red). **b,** Flow cytometry estimation of *O. fusiformis* genome size by comparison of its propidium iodide (PI)-stained nuclear DNA content against *Drosophila melanogaster*. **c,** GenomeScope 2.0 profile and *k*-mer based genome size estimation. **d,** *k*-mer distribution plot indicating the nearly complete de-haploidisation of the reference genome assembly. **e,** Smudgeplot ploidy estimation. Diploid (AB) *k*-mer pairs are predominant (0.96) for *O. fusiformis* genome, suggesting diploidy. **f,** HiC contacts map showing the inferred twelve chromosomes of *O. fusiformis*. **g,** Genome assembly and annotation statistics. **h,** Comparison of metazoan BUSCO values of selected annelid genomes. **h,** Gene and transposable element (TE) density over the inferred karyotype of *O. fusiformis*.

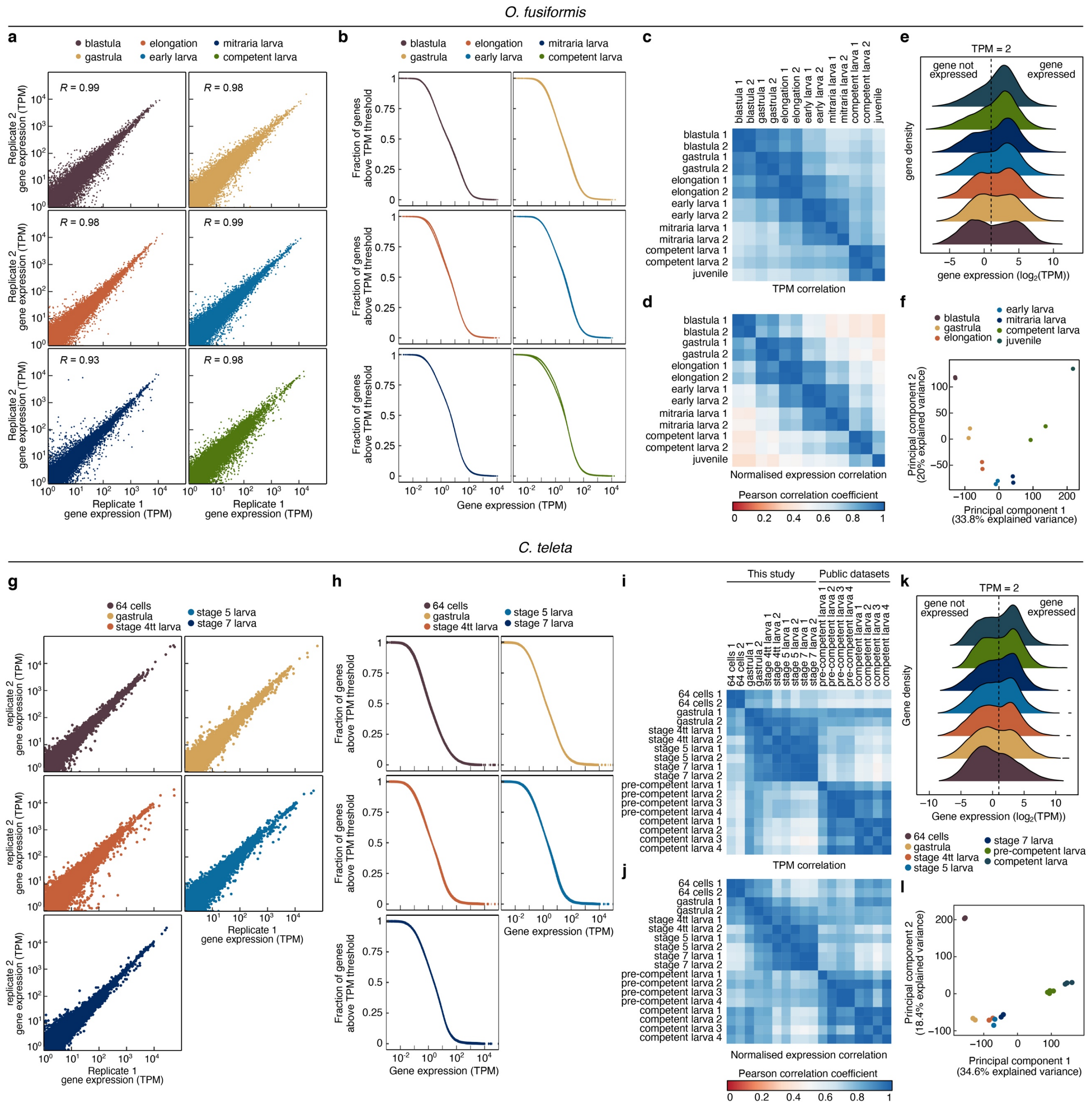

Supplementary Figure 2 | Quality control and sample correlation of stage-specific RNA-seq samples of *O. fusiformis* and *C. teleta*. **a, b,** Pairwise scatterplots (**a**) and pairwise cumulative frequency plots (**b**) of gene expression levels in transcripts per million (TPM) between biological replicates in *O. fusiformis*. *R*: Pearson correlation coefficient. **c, d,** Correlation matrices between RNA-seq experiments, calculated from a gene expression matrix in TPM (**c**) and a variance stabilising-transformed matrix of the normalised DESeq2 matrix (**d**) in *O. fusiformis*. Adequate agreement between biological replicates can be observed in both matrices. **e**, Ridgeline plots of the distribution of genes by gene expression levels in *O. fusiformis* used to experimentally defined a cut-off value of TPM = 2 to deem a gene expressed. **f,** Principal component analysis of the developmental RNA-seq time course of *O. fusiformis*. **g–l**, Equivalent plots to **a–f** for *C. teleta* RNA-seq samples.

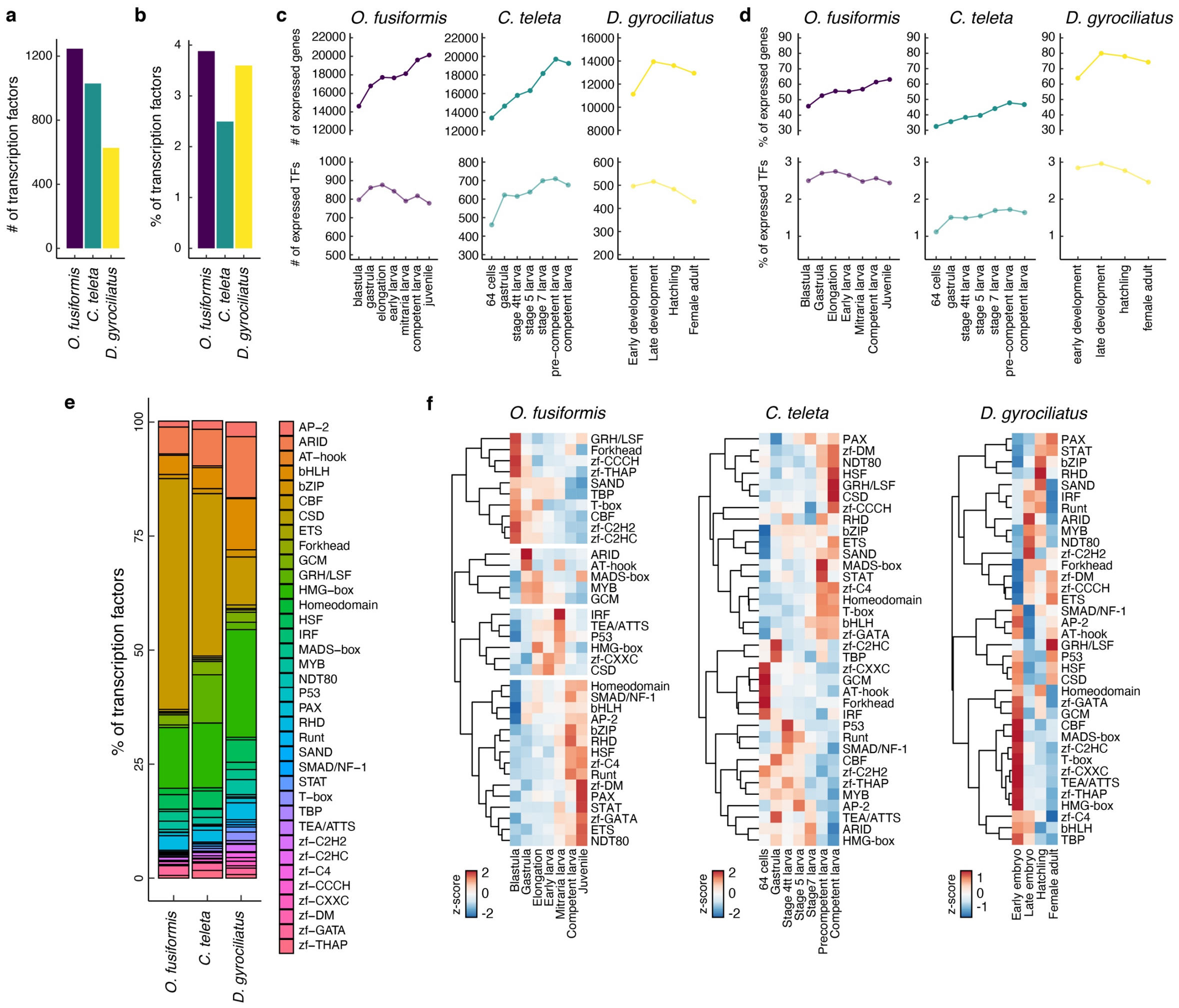

Supplementary Figure 3 | Transcription factor repertoire profiling. a, b, Absolute number of transcription factors (**a**) and their percentage relative to the whole genome (**b**) in all 3 studied annelid lineages. **c,** Number of expressed genes (top) and transcription factors (bottom) during *O. fusiformis* (left), *C. teleta* (centre) and *D. gyrociliatus* (right) development. **d,** Percentage of expressed genes (top) and transcription factors (bottom) during *O. fusiformis* (left), *C. teleta* (centre) and *D. gyrociliatus* (right) development. **e,** Barplots depicting the transcription factor repertoire in annelids by transcription factor class. **f,** Clustered heatmaps of average expression dynamics (z-score) of each transcription factor class during *O. fusiformis* (left), *C. teleta* (centre) and *D. gyrociliatus* (right) development.

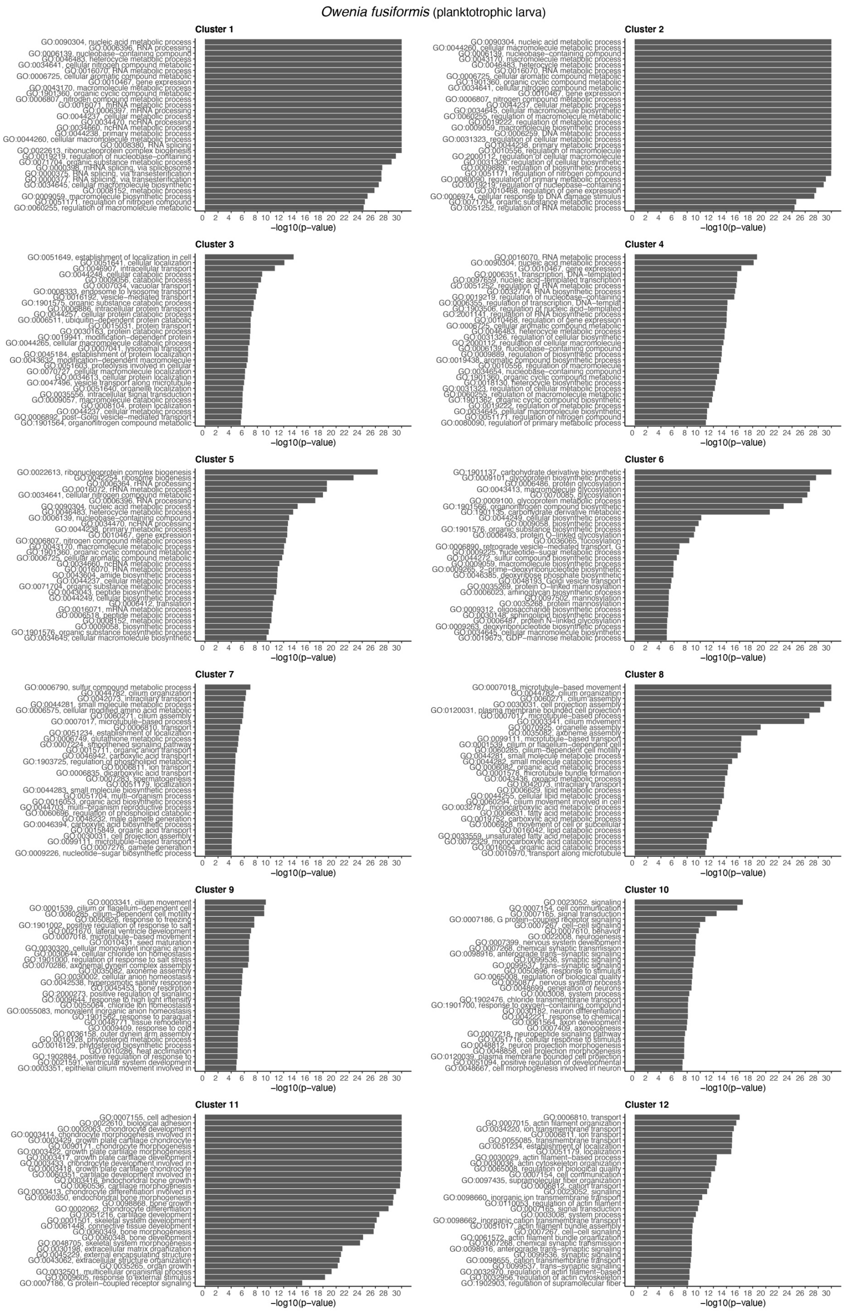

Supplementary Figure 4 | GO terms enrichment of RNA-seq clusters of *O. fusiformis*. Bar plots depicting unadjusted *p*–values of the top 30 gene ontology (GO) terms for biological process for each of the 12 clusters (1–12; left to right, top to bottom) of co-regulated transcripts retrieved through soft *k*-means clustering. *P*-values were derived from upper-tail Fisher's exact tests. Cluster 3 is the only cluster with a bimodal expression at pre- and post-larval stages.

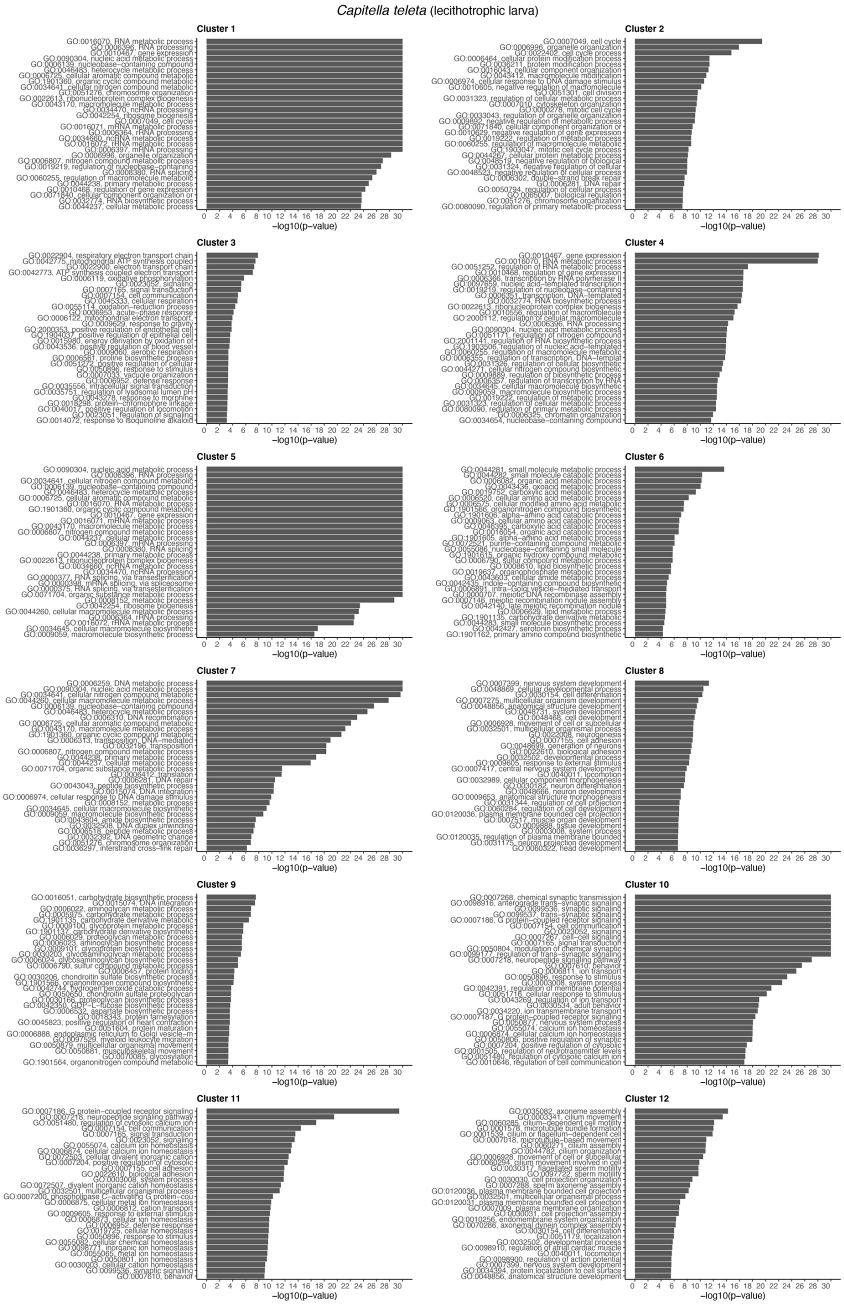

Supplementary Figure 5 | GO terms enrichment of RNA-seq clusters of *C. teleta*. Bar plots depicting unadjusted *p*–values of the top 30 gene ontology (GO) terms for biological process for each of the 12 clusters (1–12; left to right, top to bottom) of co-regulated transcripts retrieved through soft *k*-means clustering. *P*-values were derived from upper-tail Fisher's exact tests. Cluster 3 is the only cluster with a bimodal expression at pre- and post-larval stages.

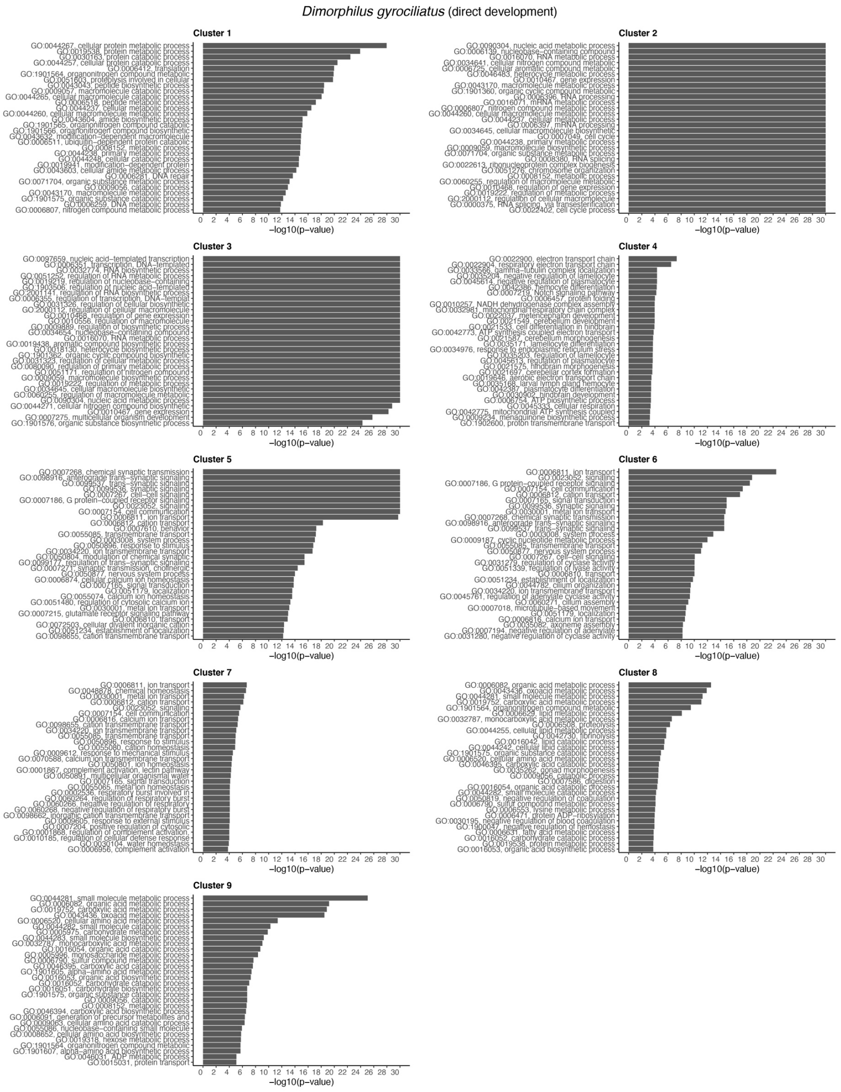

Supplementary Figure 6 | GO terms enrichment of RNA-seq clusters of *D. gyrociliatus*. Bar plots depicting unadjusted *p*–values of the top 30 gene ontology (GO) terms for biological process for each of the 9 clusters (1–9; left to right, top to bottom) of co-regulated transcripts retrieved through soft *k*-means clustering. *P*-values were derived from upper-tail Fisher's exact tests.

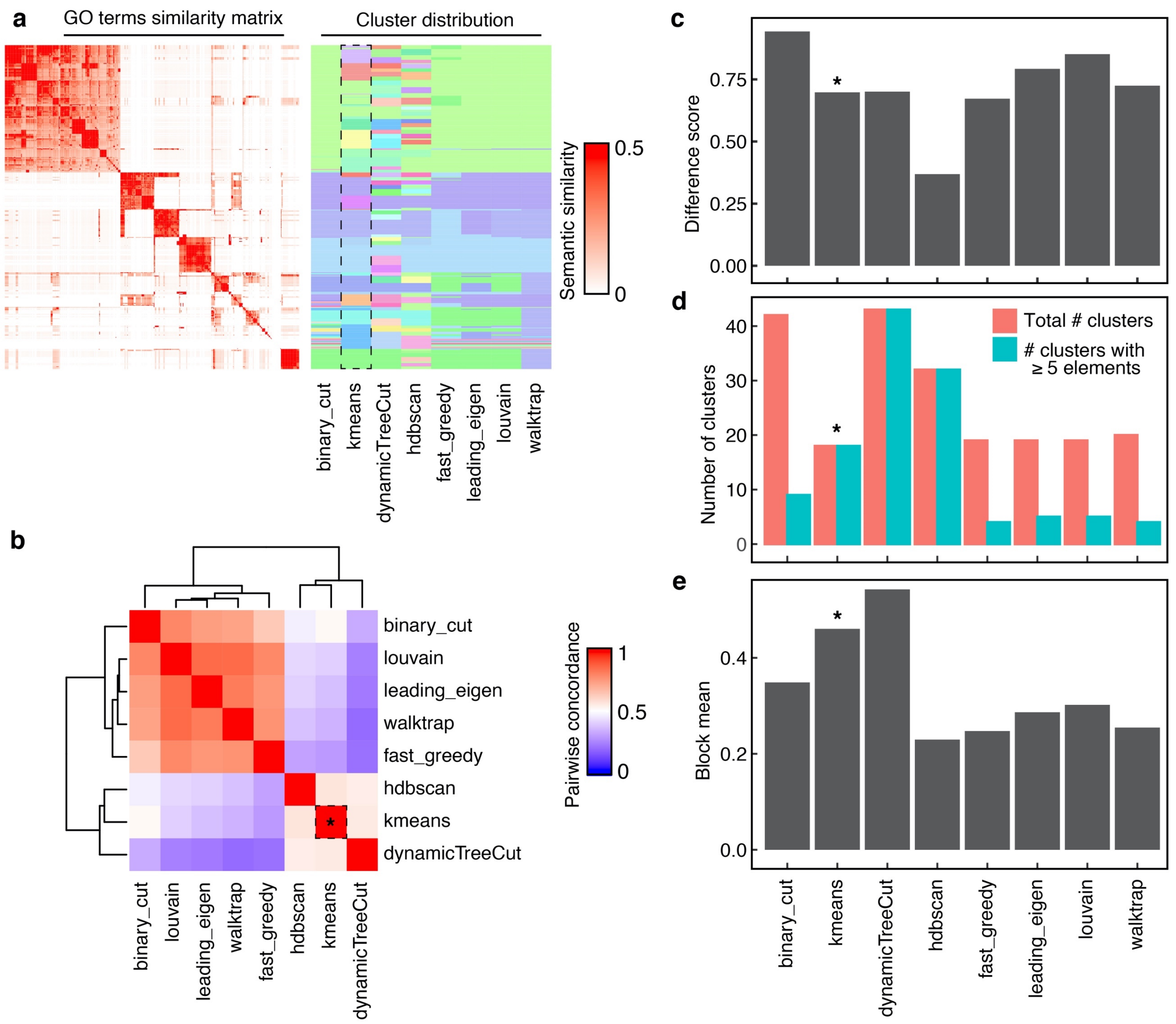

Supplementary Figure 7 | Benchmarking of clustering of GO terms enriched in annelid RNA-seq clusters. a, Gene ontology (GO) terms similarity matrix clustered by semantic similarity (left) and distribution of inferred clusters for up to 8 different clustering methods. **b,** Correlation matrix between clustering methods. **c,** Distribution difference score between vectors $\mathbf{x_1}$ and $\mathbf{x_2}$ for each clustering method, where $\mathbf{x_1}$ contains the semantic similarities of all pairs of elements belonging to the same cluster, and $\mathbf{x_2}$ the similarity of all pairs of elements belonging to different clusters, calculated as the two-tailed Kolmogorov-Smirnov statistic. **d,** Total number of clusters (pink) and number of clusters populated with at least 5 GO terms (blue) for each clustering method. **e,** Mean similarity score for each block/cluster, calculated as the mean of vector $\mathbf{x_1}$. *k*-means clustering (denoted by the dotted lines and the asterisks) was chosen for displaying an even distribution of clusters (**a**) populated with sufficiently similar elements (**c, e**), and for rendering the smallest number of clusters where every cluster is at least comprised of 5 GO terms (**d**).

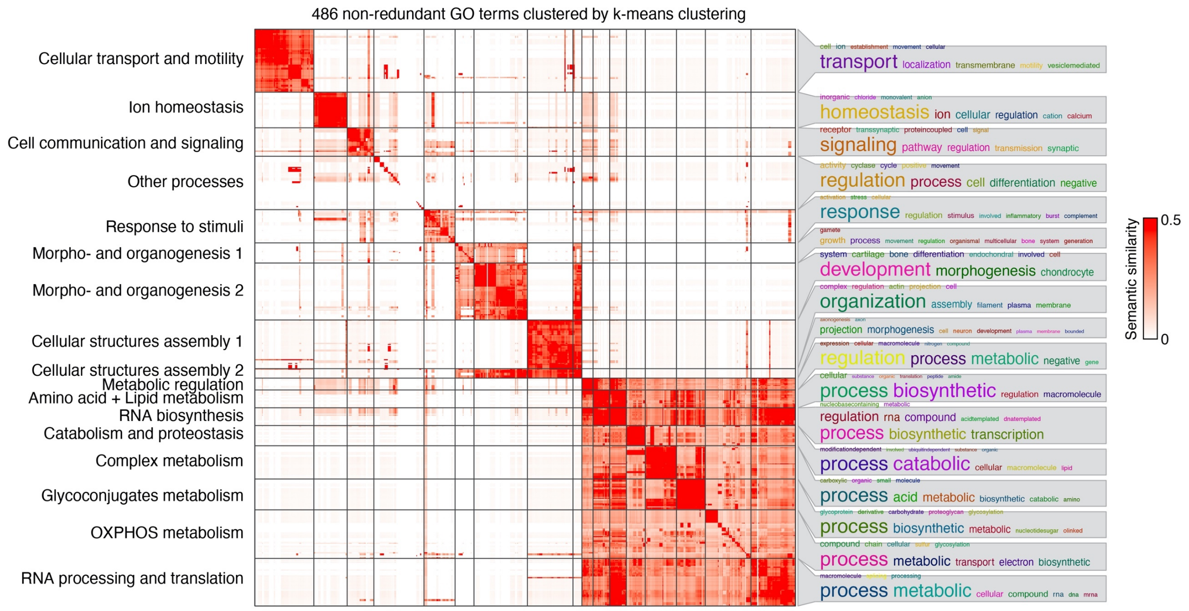

Supplementary Figure 8 | Clustering of GO terms enriched in annelid RNA-seq clusters. Similarity matrix of all 486 non-redundant gene ontology (GO) terms for biological process from the GO term enrichment analysis performed on annelid RNA-seq clusters. GO terms were clustered through *k*-means clustering according to their semantic similarity (associated clustering benchmarking is explained in Supplementary Figure 7). Word clouds recapitulate each cluster, with word size being proportional to word frequency in the GO terms contained within that cluster. Custom umbrella terms were selected to summarise each cluster of GO terms, here shown on the left of the matrix.

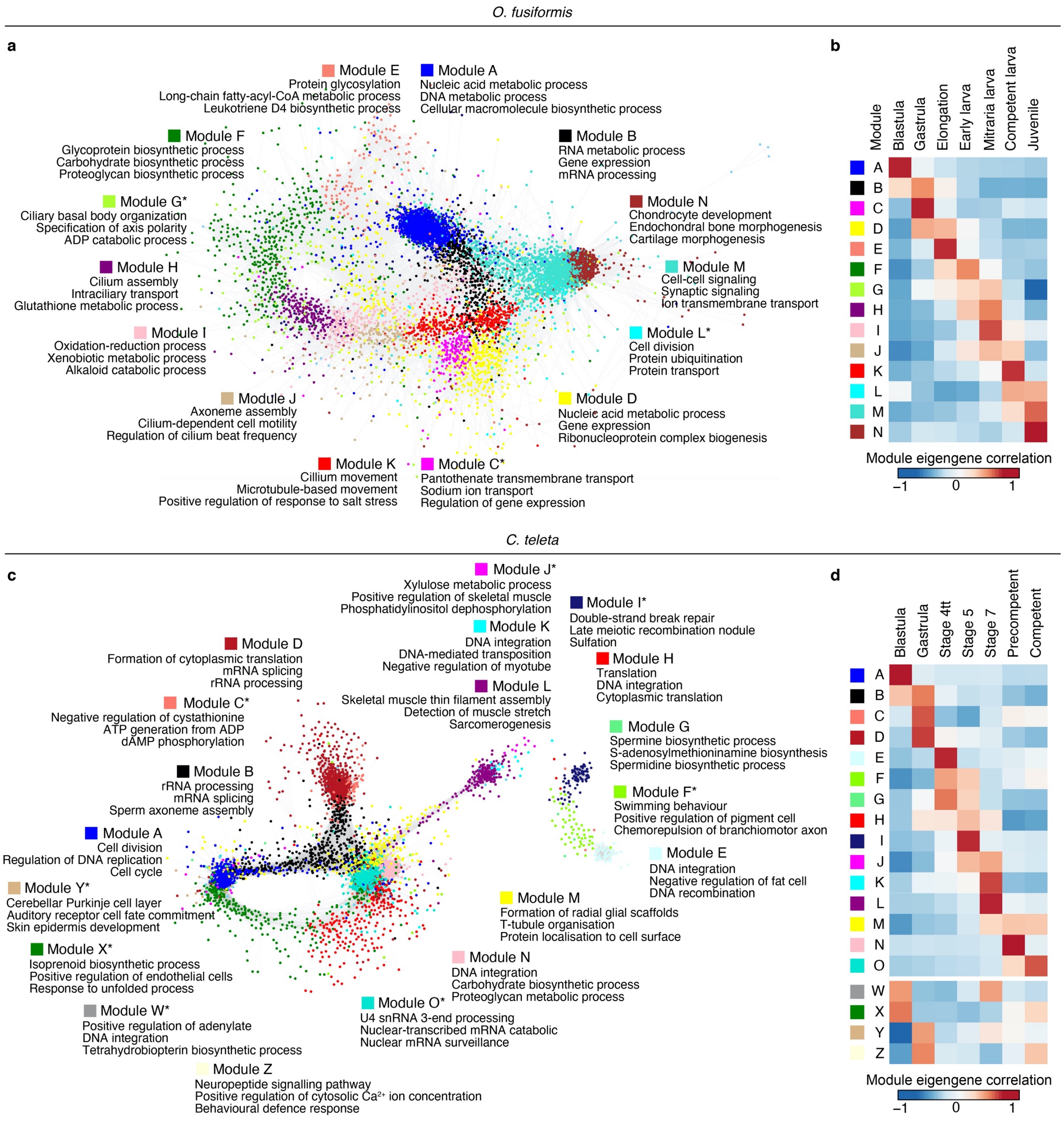

Supplementary Figure 9 | Weighted gene co-expression network analyses. a, c, Force-directed layout representations of the weighted gene co-expression network analyses (WGCNA) of *O. fusiformis* (**a**) and *C. teleta* (**b**). For visualisation purposes, only the nodes and edges of a random selection of 30% of the transcripts of both species are depicted here. For each of the retrieved co-expression modules, representative enriched GO terms are shown. Full lists are listed in Supplementary Figures 10 and 11 for *O. fusiformis* and *C. teleta*, respectively. Unadjusted *p*–values of GO terms from modules flagged with an asterisk (*) were lower than average. **b, d,** WGCNA module eigengene correlation with each developmental stage of *O. fusiformis* (**b**) and *C. teleta* (**d**). Modules named as W, X, Y, and Z for *C. teleta* are correlated with non-consecutive developmental stages unlike all other modules and are therefore depicted separately. Gene modules obtained through the WGCNA approach for both species support and complement our soft clustering method.

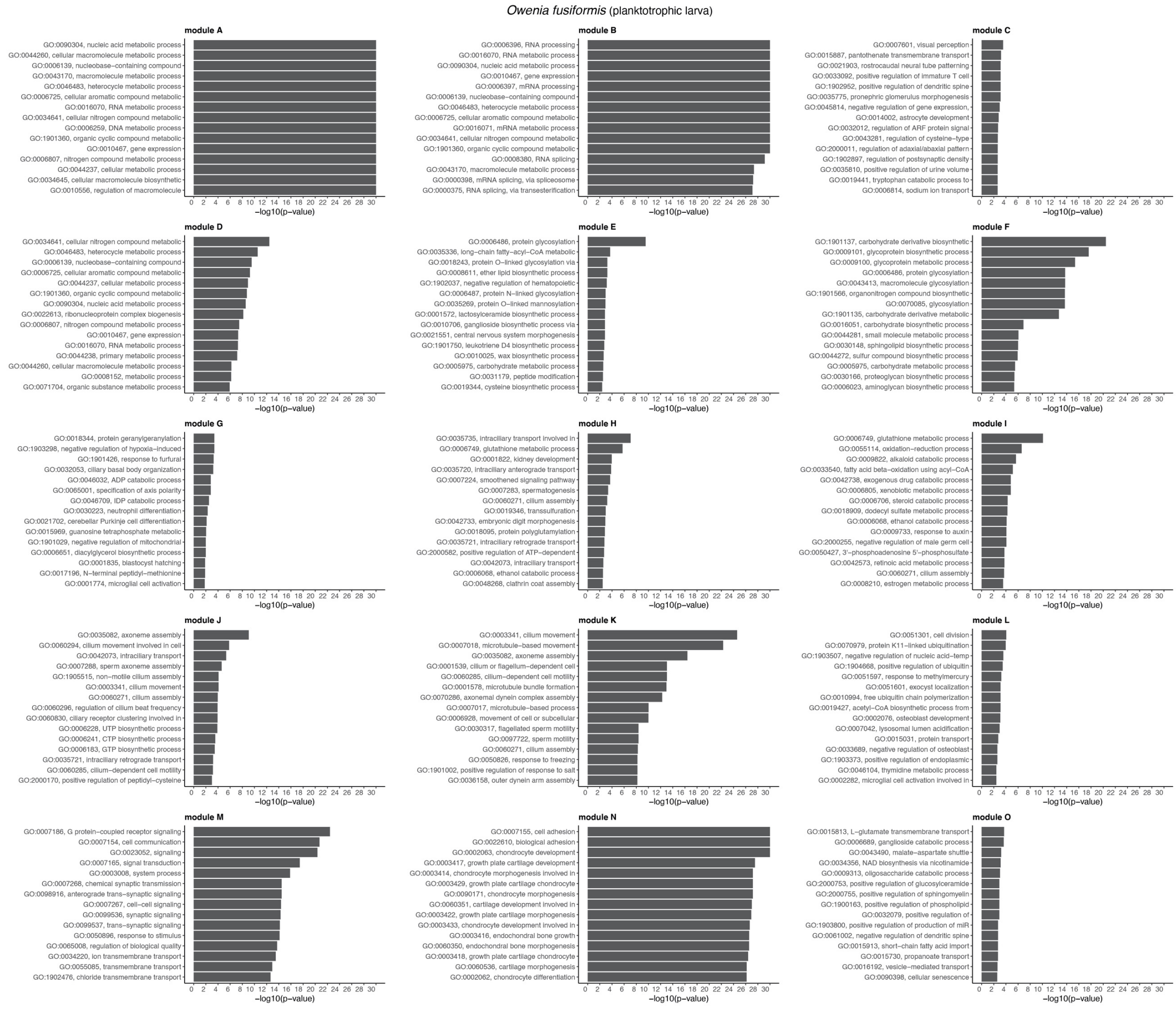

Supplementary Figure 10 | GO terms enrichment of WGCNA modules of *O. fusiformis*. Bar plots depicting unadjusted *p*–values of the top 15 gene ontology (GO) terms for biological process for each of the 15 modules (A–O; left to right, top to bottom) of co-expressed transcripts retrieved through our WGCNA approach. *P*-values were derived from upper-tail Fisher's exact tests.

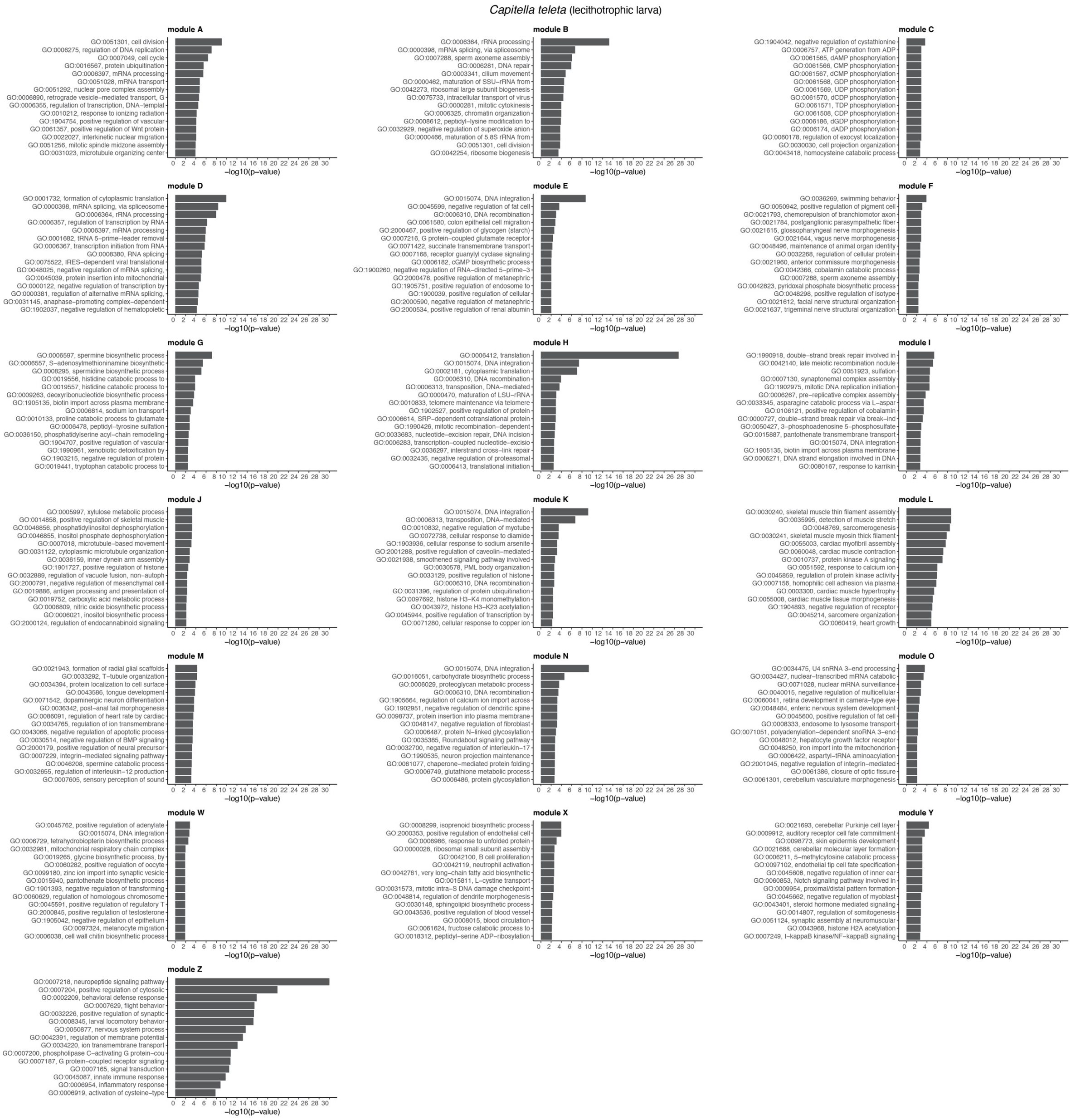

Supplementary Figure 11 | GO terms enrichment of WGCNA modules of *C. teleta*. Bar plots depicting unadjusted *p*–values of the top 15 gene ontology (GO) terms for biological process for each of the 19 modules (A–O, and W–Z; left to right, top to bottom) of co-expressed transcripts retrieved through our WGCNA approach. *P*-values were derived from upper-tail Fisher's exact tests.

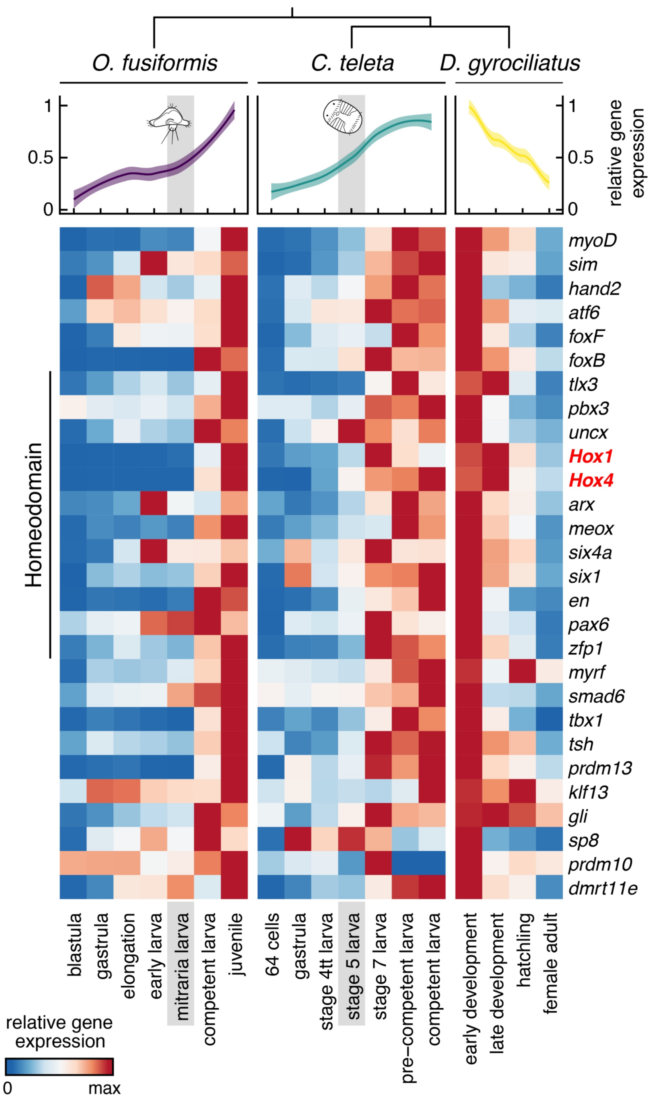

Supplementary Figure 12 | Expression dynamics of transcription factors consistently under heterochronic shifts between indirect and direct development. Heatmaps of gene-wise (bottom) expression, and average expression dynamics (top) during *O. fusiformis*, *C. teleta* and *D. gyrociliatus* development of transcription factors consistently shifted from late expression across both species with indirect development to early expression in *D. gyrociliatus*. Gene symbols are depicted on the right side of the heatmap, with those belonging to the Homeodomain transcription factor class highlighted on the left side of the heatmap. Curves in the top are locally estimated scatterplot smoothings, coloured shaded areas represent standard error of the mean.

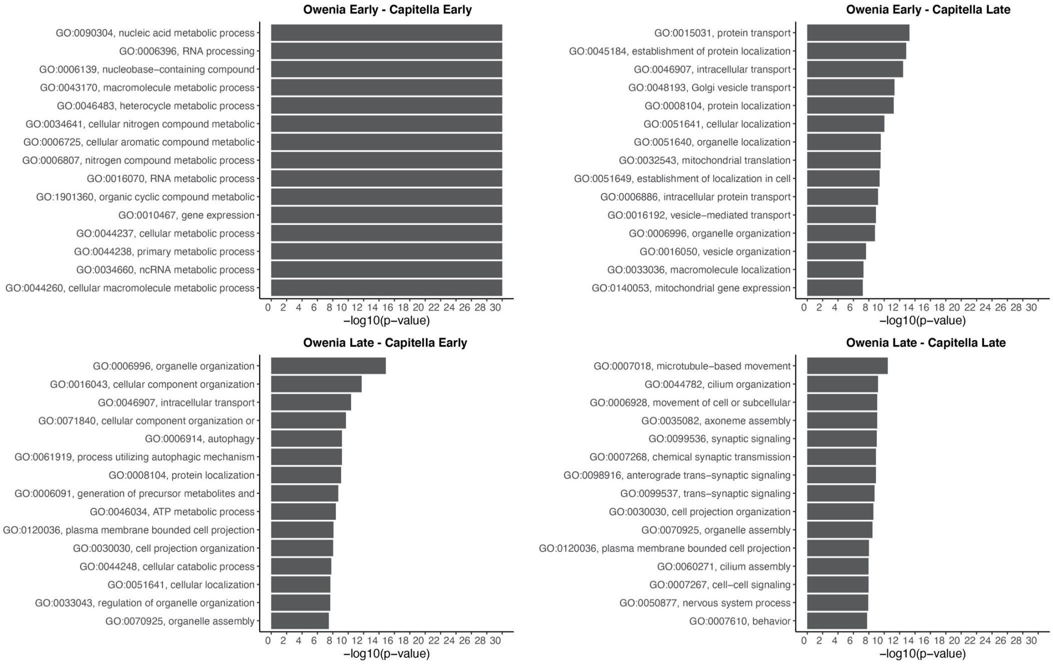

Supplementary Figure 13 | GO terms enrichment of gene sets with different timings of expression between *O. fusiformis* and *C. teleta*. Bar plots depicting unadjusted *p*–values of the top 15 gene ontology (GO) terms for biological process for each of the 4 single copy ortholog gene sets between *O. fusiformis* and *C. teleta* with different timings of expression. Genes with similar expression dynamics, expressed either in early or pre-larval clusters (top left) or in late or post-larval clusters (bottom right) in both species, are opposed to gene sets under heterochronic shifts, either expressed early in *O. fusiformis* and late in *C. teleta* ("delayed", top right) or late in *O. fusiformis* and early in *C. teleta* ("accelerated", bottom left). *P*-values were derived from upper-tail Fisher's exact tests.

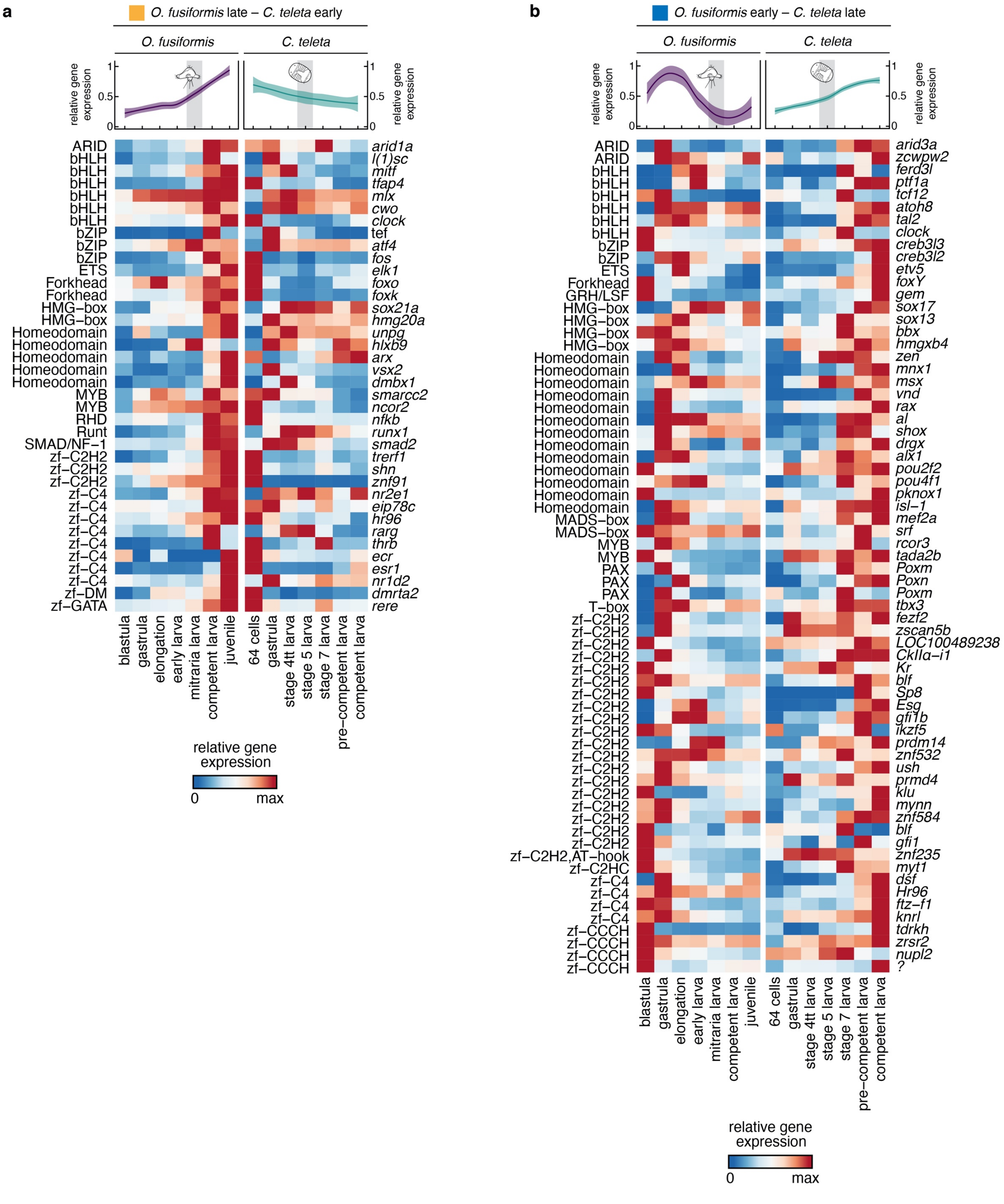

Supplementary Figure 14 | Expression dynamics of transcription factors under heterochronic shifts between larva types. a, b, Heatmaps of gene-wise (bottom) expression, and average expression dynamics (top) during *O. fusiformis* and *C. teleta* development of transcription factors shifted from late expression in *O. fusiformis* to early expression in *C. teleta* ("accelerated", **a**) or vice versa ("delayed", **b**). Gene symbols are depicted on the right side of the heatmap, with transcription factor class for each gene is shown on the left side. Curves are locally estimated scatterplot smoothings, coloured shaded areas represent standard error of the mean.

a

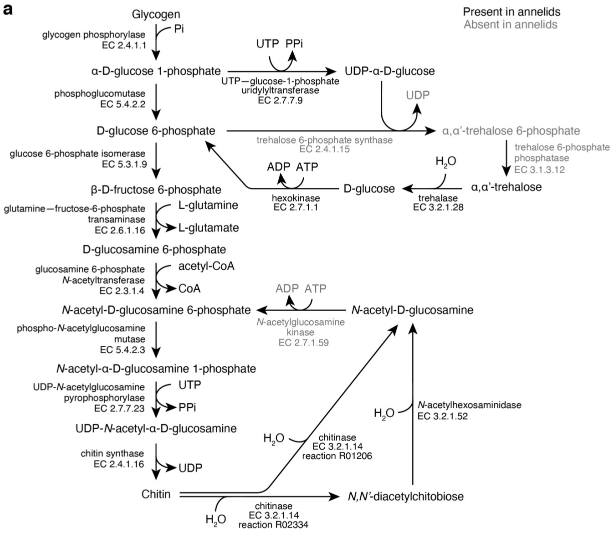

b

Enzymatic activity	*S. cerervisiae* genes	*D. melanogaster* genes
glycogen phosphorylase	GPH1	GlyP
phosphoglucomutase	PGM1, PGM2	Pgm2a, Pgm2b
UTP glucose-1-phosphate uridylyltransferase	UGP1	UGP
trehalose 6-phosphate synthase	TPS1	Tps1
trehalose 6-phosphate phosphatase	TPS2	CG5171
trehalase	ATH1, NTH1, NTH2	Treh
hexokinase	HXK1, HXK2, EMI2, GLK1, YLR4	Hex-A, Hex-C, Hex-t1, Hex-t2
glucose 6-phosphate isomerase	PGI1	Pgi
glutamine—fructose-6-phosphate isomerase	GFA1	Gfat1, Gfat2
glucosamine 6-phosphate *N*-acetyltransferase	GNA1	Gnpnat
phospho N-acetylglucosamine mutase	PCM1	nst
UDP-*N*-acetylglucosamine pyrophosphorylase	QRI1	mmy
chitin synthase	CHS1, CHS2, CHS3	kkv, Chs2
chitinase	CTS1, CTS2	Cht2, Cht4, Cht5, Cht6, Cht7, Cht8 Cht9, Cht10, Cht11, Cht12, LysD
N-acetylhexosaminidase	–	Hexo1, Hexo2, NaDg, fdl, Oga, CG7985
N-acetylglucosamine kinase	–	Nagk

c

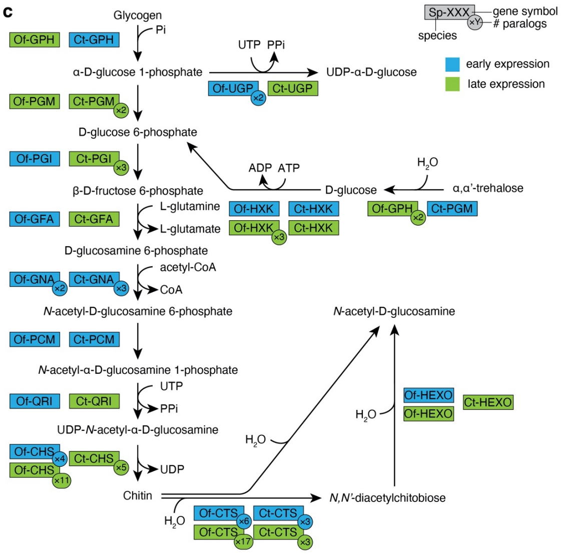

d

Enzymatic activity	Heterochronic shift across species	Shift completeness
glycogen phosphorylase	*O. fusiformis* late – *C. teleta* early	Complete
phosphoglucomutase	–	–
UTP glucose-1-phosphate uridylyltransferase	*O. fusiformis* early – *C. teleta* late	Complete
trehalase	*O. fusiformis* late – *C. teleta* early	Complete
hexokinase	–	–
glucose 6-phosphate isomerase	*O. fusiformis* early – *C. teleta* late	Complete
glutamine—fructose-6-phosphate isomerase	*O. fusiformis* early – *C. teleta* late	Complete
glucosamine 6-phosphate *N*-acetyltransferase	–	–
phospho N-acetylglucosamine mutase	–	–
UDP-*N*-acetylglucosamine pyrophosphorylase	*O. fusiformis* early – *C. teleta* late	Complete
chitin synthase	*O. fusiformis* early – *C. teleta* late	Partial
chitinase	–	–
N-acetylhexosaminidase	*O. fusiformis* early – *C. teleta* late	Partial

Supplementary Figure 15 | Heterochronic shift of the annelid chitin synthesis pathway genes between larval types. a, Schematic depiction of the canonical chitin synthesis pathway in insects and fungi and our reconstruction of the annelid pathway. Enzymatic activities missing in annelids and their cofactors are shown in grey. Enzyme Commission (EC) numbers are shown for all enzymatic activities. KEGG reaction R numbers are depicted for promiscuous enzymatic activities. ADP: adenosine diphosphate; ATP: adenosine triphosphate; CoA: coenzyme A; Pi: inorganic phosphate; PPi: pyrophosphate; UDP: uridine diphosphate; UTP: uridine triphosphate. **b,** *Saccharomyces cerevisiae* and *Drosophila melanogaster* genes with enzymatic activities involved in the chitin synthesis pathway, highlighting the high number of paralogs within species for many activities (e.g., phosphoglucomutase, hexokinase, chitinase, *N*-acetylhexosaminidase, etc.) and the potential problems with single copy ortholog strategies for orthology assignment. **c,** Expression timing (blue: early, pre-larval; green: late, post-larval) of the PANTHER ID-inferred chitin synthesis genes in *O. fusiformis* and *C. teleta*. Paralog number for each expression timing is shown next to the gene symbols. Ct: *C. teleta*; Of: *O. fusiformis*. **d,** Summary of enzymatic activities under heterochronic shift between *O. fusiformis* and *C. teleta*, and whether the shift involves all paralogs for the activity (complete shift) or a limited number only (partial shift). A total of 46 % of enzymatic activities are expressed early in *O. fusiformis* and late in *C. teleta*, with only 15 % in the opposite direction. These results align with the earlier development of chaetae in planktotrophic larvae like the one from *O. fusiformis*.

KEGG map04140: Animal autophagy pathway

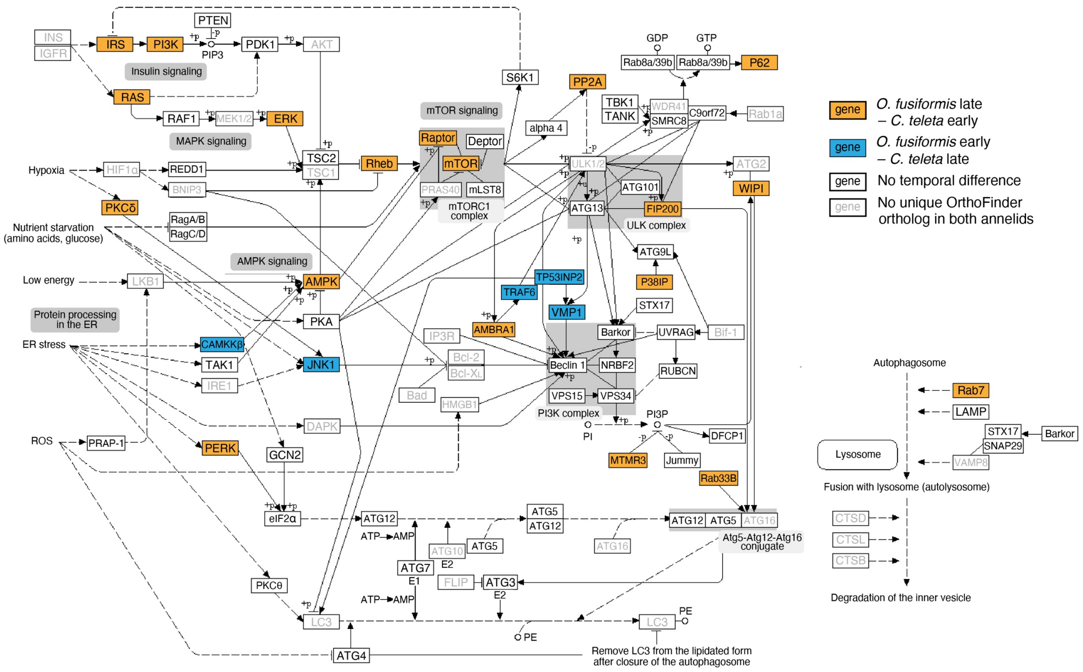

Supplementary Figure 16 | Heterochronic shift of the autophagy pathway genes between larval types. Schematic depiction of the animal autophagy pathway (KEGG: map04140). A total of 80 % of the genes under heterochronic shifts (19 of 24) are displaced from post-larval expression in *O. fusiformis* to pre-larval expression in *C. teleta*. These results align with the early need for yolk consumption in lecithotrophic larvae like the one from *C. teleta*.

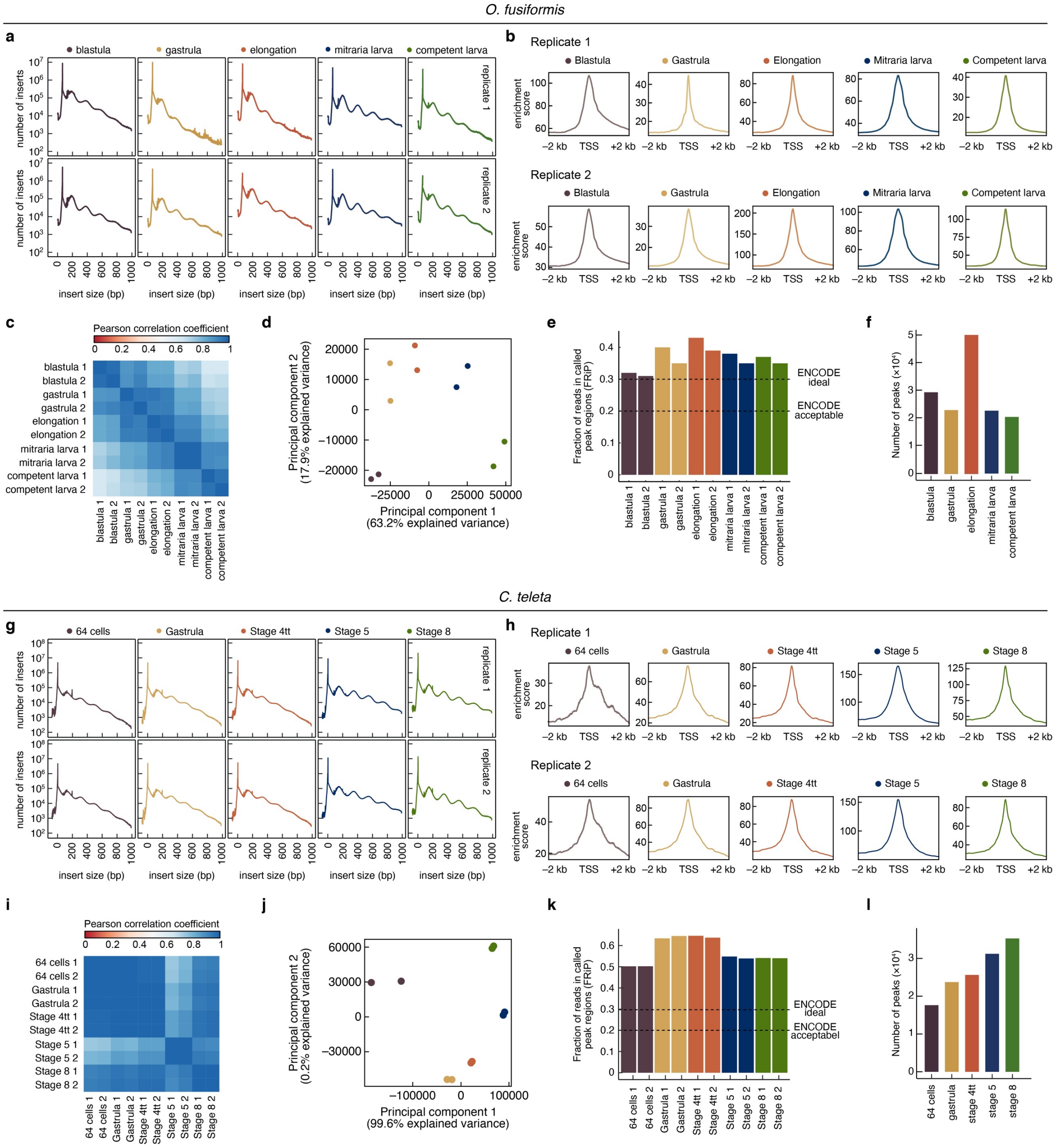

Supplementary Figure 17 | Quality control and sample correlation of stage-specific ATAC-seq samples of *O. fusiformis* and *C. teleta*. **a,** Insert size distribution of ATAC-seq libraries of *O. fusiformis*. Nucleosomal ladders are visible for all samples. **b,** Summary plots of ATAC-seq enrichment around transcription start sites (TSS; ±2 kb) in *O. fusiformis.* These demonstrate at least a two-fold enrichment for all samples. **c,** Correlation matrix based on peak accessibility of the called consensus ATAC-seq peak set for *O. fusiformis*. **d,** Principal component analysis of *O. fusiformis* ATAC-seq samples based on peaks as in (**c**). **e,** FRiP scores for *O. fusiformis* ATAC-seq samples indicating acceptable (FRiP = 0.2) and ideal (FRiP = 0.3) values as per ENCODE standards (dotted lines). **f,** Bar plot of ATAC-seq peak number by developmental stage for *O. fusiformis*. **g–l**, Equivalent plots to **a–f** for *C. teleta* ATAC-seq samples.

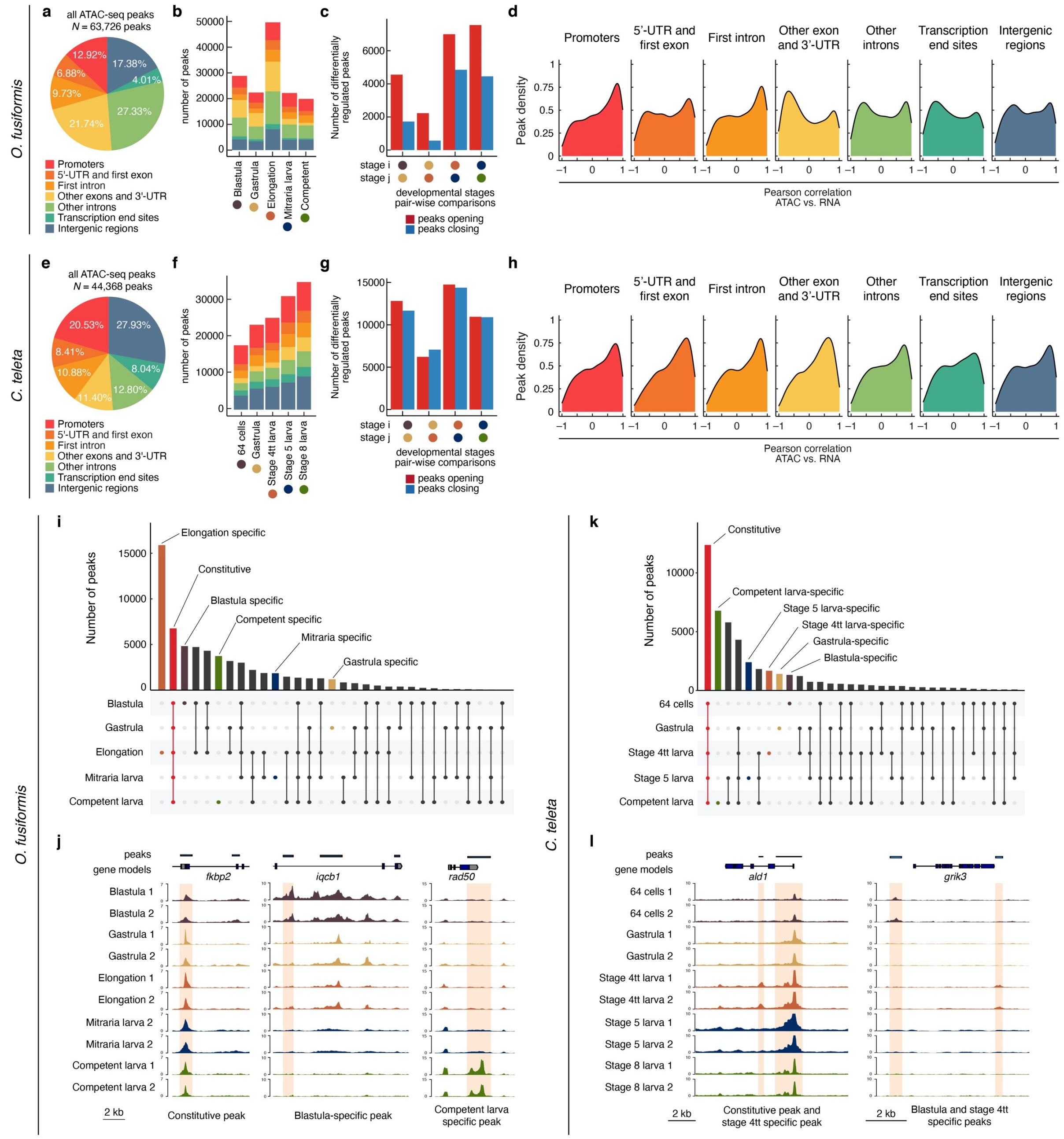

Supplementary Figure 18 | Accessible chromatin landscape profiling. a, Genomic feature annotation of the called consensus ATAC-seq peak set for *O. fusiformis*. **b,** Stacked bar plot of ATAC-seq peak number by developmental stage for *O. fusiformis*, classified by genomic feature. **c,** Differentially accessible peaks for all four pair-wise comparisons between consecutive developmental stages in *O. fusiformis*. Only significant differentially regulated peaks (Benjamini-Hochberg-adjusted *p*–value < 0.05 obtained using the described DESeq2 pipeline) are shown. **d,** Density plots based on the Pearson correlation coefficient between ATAC-seq peak accessibility and RNA-seq expression level of the nearest gene/transcript to the ATAC-seq peak in *O. fusiformis*. Peaks in promoters and first introns are the most positively correlated with gene expression, with most other genomic regions displaying a bimodal distribution. **e**–**h**, Equivalent plots to **a**–**d** for *C. teleta*. Unlike in *O. fusiformis*, accessible chromatin regions in *C. teleta* mostly only correlate positively with gene expression, regardless of the genomic feature. **i,** UpSet plot classification of the ATAC-seq peaks of *O. fusiformis* by developmental stage. Peaks present in more than one stage are coloured in

grey. **j,** Representative views of constitutive and stage-specific peaks for *O. fusiformis*, with the ATAC-seq peak track indicating the consensus ATAC-sea peak set. *fkbp2*: FKBP prolyl isomerase 2; *iqcb1*: IQ calmodulin-binding motif-containing protein 1; *rad50*: RAD50 double strand break repair protein. **k, l** Equivalent plots to **i, j** for *C. teleta*. *ald1*: aldolase 1; *grik3*: glutamate ionotropic receptor kainate type subunit 3.

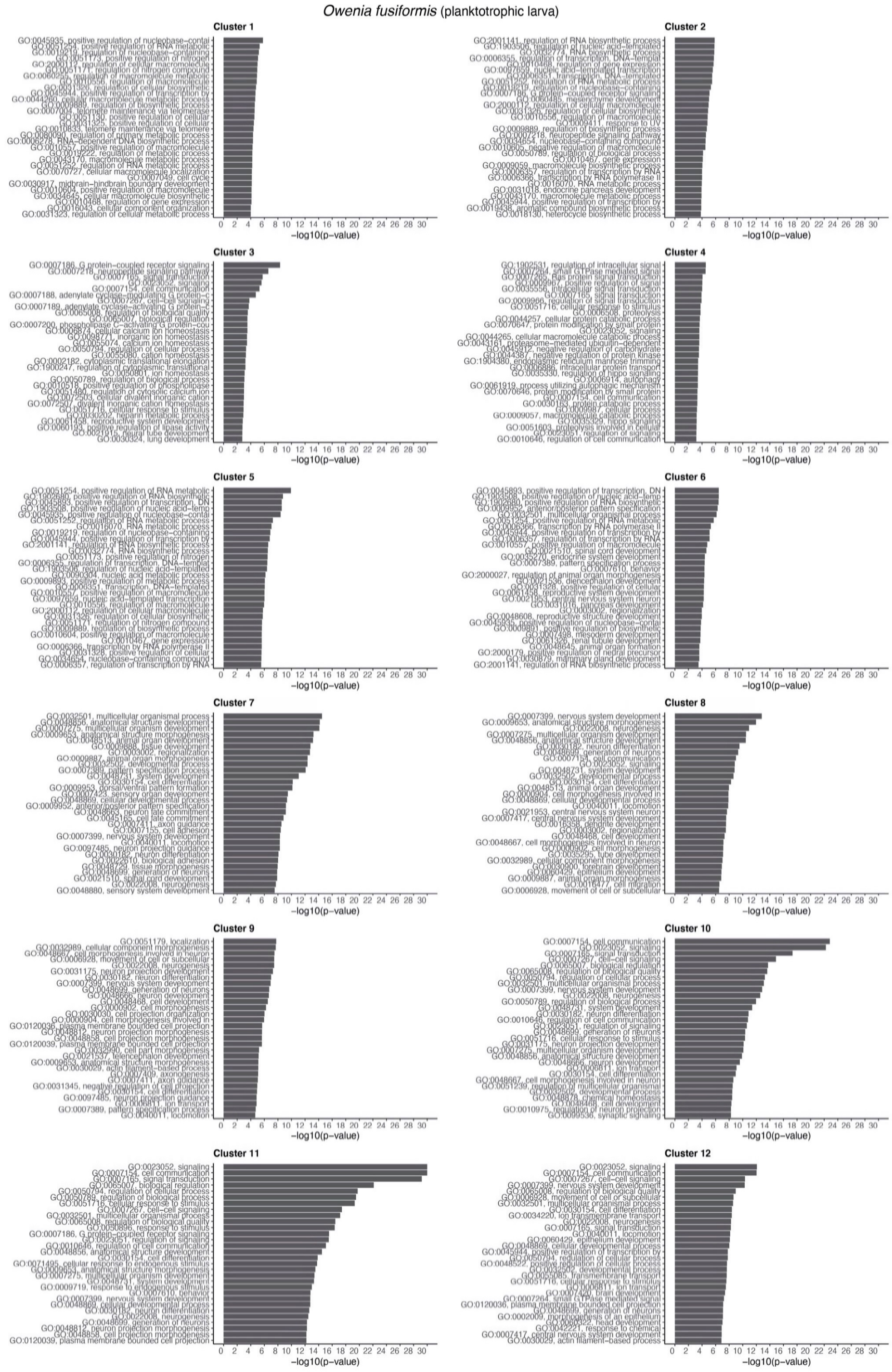

Supplementary Figure 19 | GO terms enrichment of gene sets regulated by ATAC-seq peak clusters in *O. fusiformis*. Bar plots depicting unadjusted *p*–values of the top 30 gene ontology (GO) terms for biological process for each of the gene sets regulated by each of the 12 clusters (1–12; left to right, top to bottom) of co-regulated ATAC-seq consensus peaks retrieved through soft *k*-means clustering. *P*-values were derived from upper-tail Fisher's exact tests.

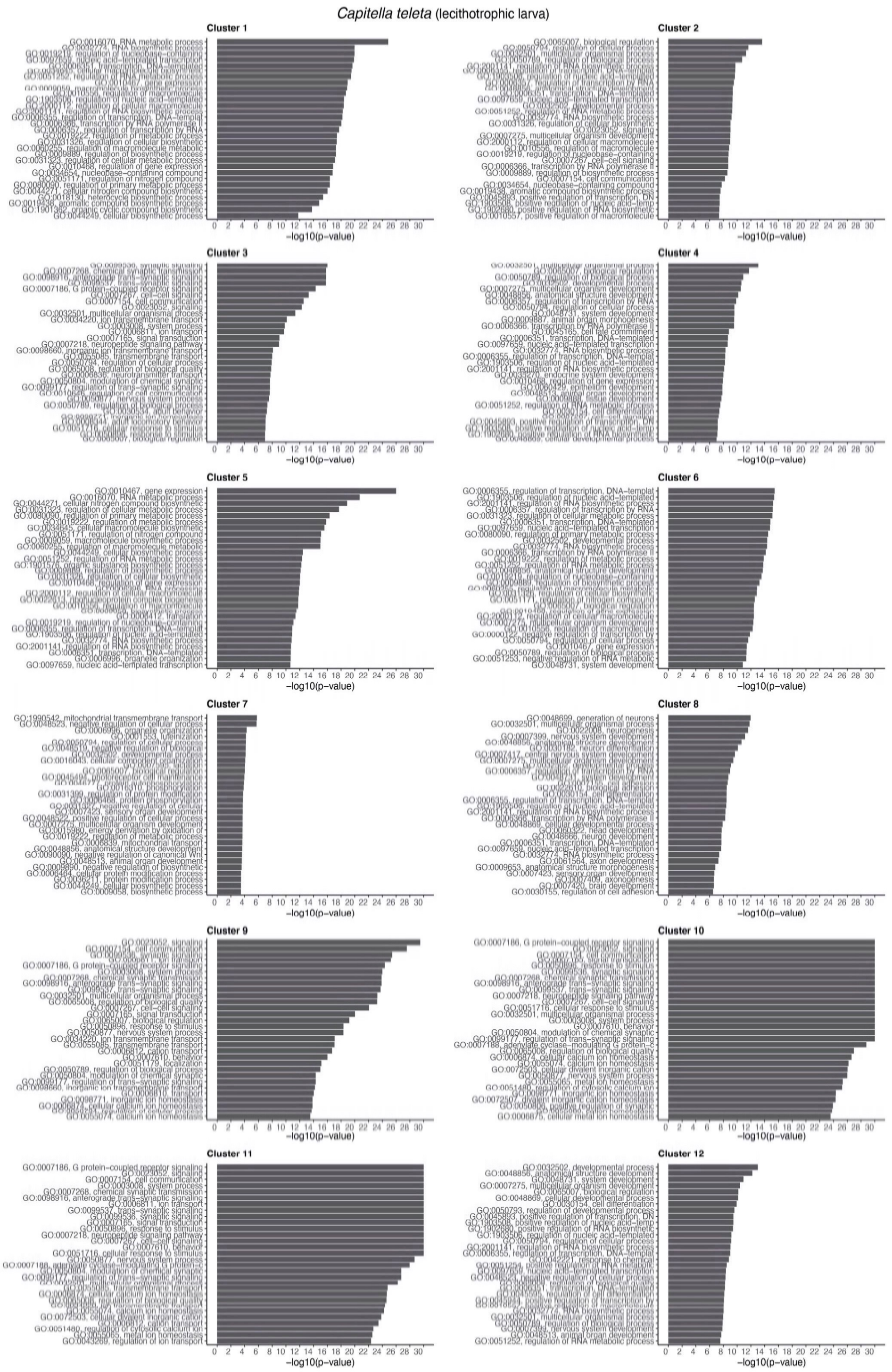

Supplementary Figure 20 | GO terms enrichment of gene sets regulated by ATAC-seq peak clusters in *C. teleta*. Bar plots depicting unadjusted *p*–values of the top 30 gene ontology (GO) terms for biological process for each of the gene sets regulated by each of the 12 clusters (1–12; left to right, top to bottom) of co-regulated ATAC-seq consensus peaks retrieved through soft *k*-means clustering. *P*-values were derived from upper-tail Fisher's exact tests.

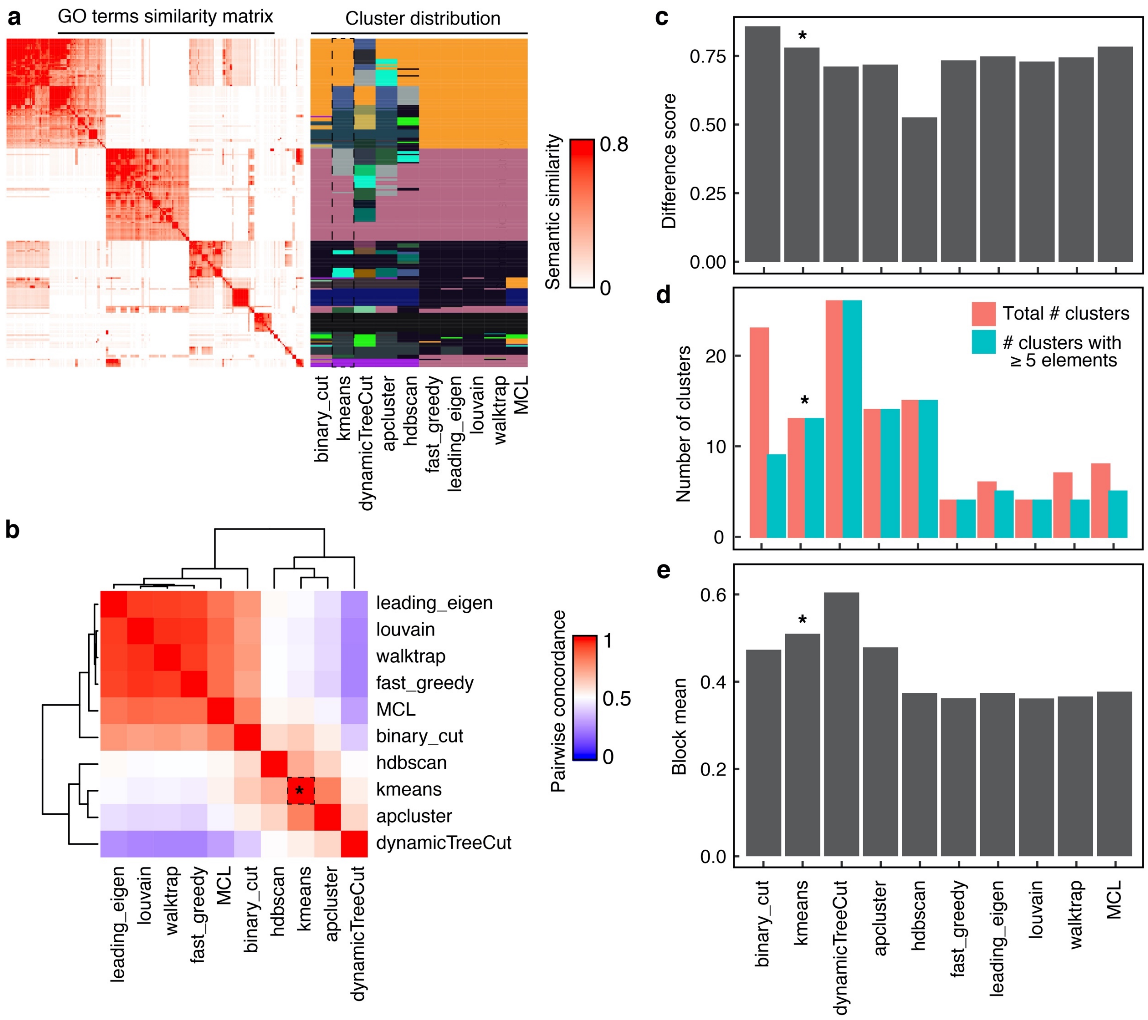

Supplementary Figure 21 | Benchmarking of clustering of GO terms enriched in gene sets regulated by annelid ATAC-seq peak clusters. a, Gene ontology (GO) terms similarity matrix clustered by semantic similarity (left) and distribution of inferred clusters for up to 10 different clustering methods. **b,** Correlation matrix between clustering methods. **c,** Distribution difference score between vectors $\mathbf{x_1}$ and $\mathbf{x_2}$ for each clustering method, where $\mathbf{x_1}$ contains the semantic similarities of all pairs of elements belonging to the same cluster, and $\mathbf{x_2}$ the similarity of all pairs of elements belonging to different clusters, calculated as the two-tailed Kolmogorov-Smirnov statistic. **d,** Total number of clusters (pink) and number of clusters populated with at least 5 GO terms (blue) for each clustering method. **e,** Mean similarity score for each block/cluster, calculated as the mean of vector $\mathbf{x_1}$. *k*-means clustering (denoted by the dotted lines and the asterisks) was chosen for displaying an even distribution of clusters (**a**) populated with sufficiently similar elements (**c, e**), and for rendering a similar number of clusters as retrieved during clustering of GO terms enriched in annelid RNA-seq clusters (see Supplementary Figures 7 and 8), where every cluster is at least comprised of 5 GO terms (**d**).

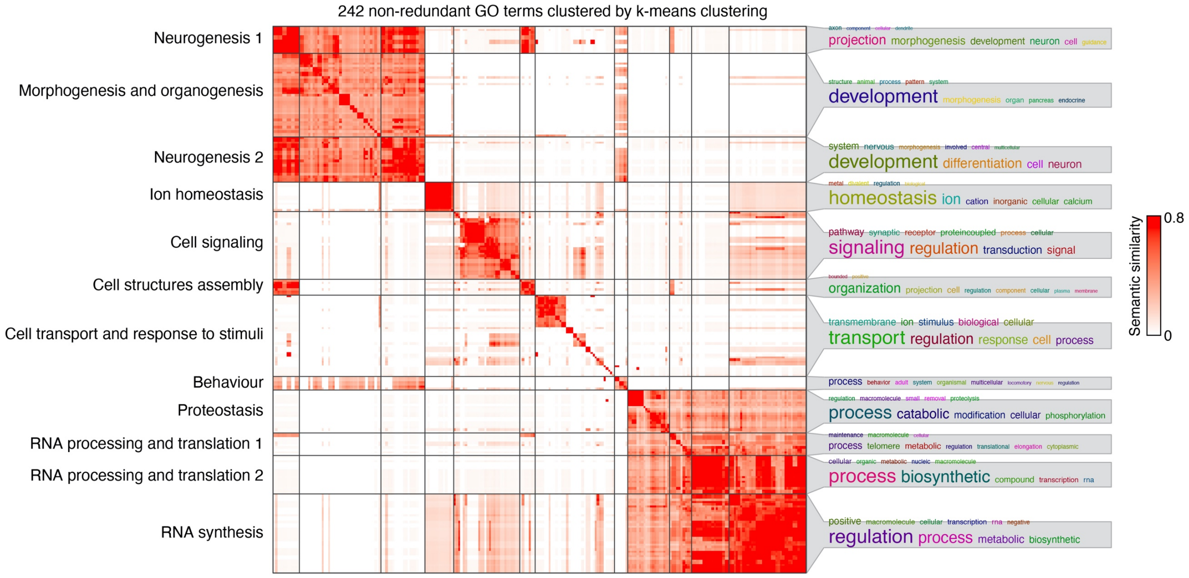

Supplementary Figure 22 | Clustering of GO terms enriched in gene sets regulated by annelid ATAC-seq peak clusters. Similarity matrix of all 242 non-redundant gene ontology (GO) terms for biological process from the GO term enrichment analysis performed on gene sets regulated by annelid ATAC-seq peak clusters. GO terms were clustered through *k*-means clustering according to their semantic similarity (associated clustering benchmarking is explained in Supplementary Figure 21). Word clouds recapitulate each cluster, with word size being proportional to word frequency in the GO terms contained within that gene set. Custom umbrella terms were selected to summarise each cluster of GO terms, here shown on the left of the matrix.

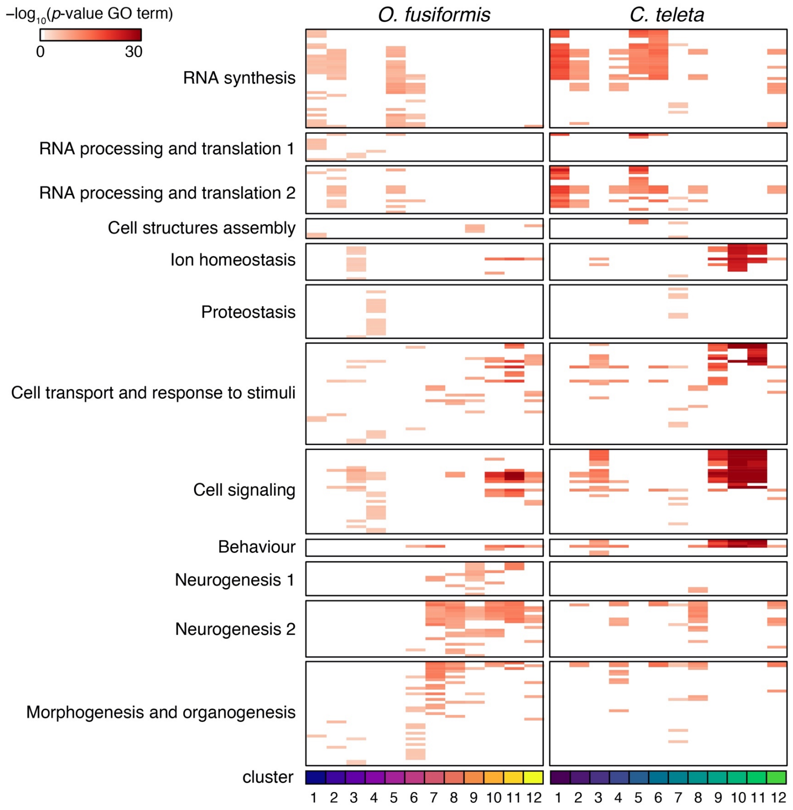

Supplementary Figure 23 | Annelid time-course of biological processes regulated by accessible chromatin. Enrichment analysis of biological process gene ontology (GO) terms for *O fusiformis* (left) and *C. teleta* (right) gene sets regulated by ATAC-seq peak clusters. Each line represents a single GO term, for which the $-\log_{10}$(*p*–value) for each gene set is shown in a colour coded scale. Clusters are shown on the bottom of the heatmap. *P*-values were derived from upper-tail Fisher's exact tests.

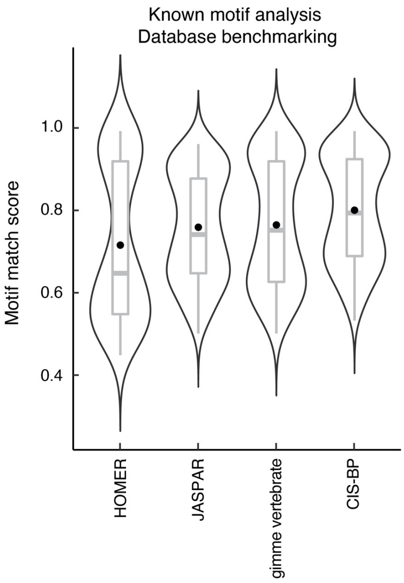

Supplementary Figure 24 | Cross-database known motif analysis benchmarking. Violin plots and boxplots depicting the distribution of known motif match scores of the full set of common annelid and species-specific clustered motif archetypes (n = 141) against HOMER, JASPAR, gimme vertebrate and CIS-BP. The pan-eukaryote CIS-BP database performs better on average, yet many of the hits belong to phylogenetically distant clades such as fungi or plants, while HOMER apparently performs poorly in comparison as a result of a lower false positive rate. For boxplots, centre lines, median; box, interquartile range (IQR); whiskers, first or third quartile ± 1.5 × IQR. Manual curation of clustered motif archetypes can be found in Supplementary Table 74.

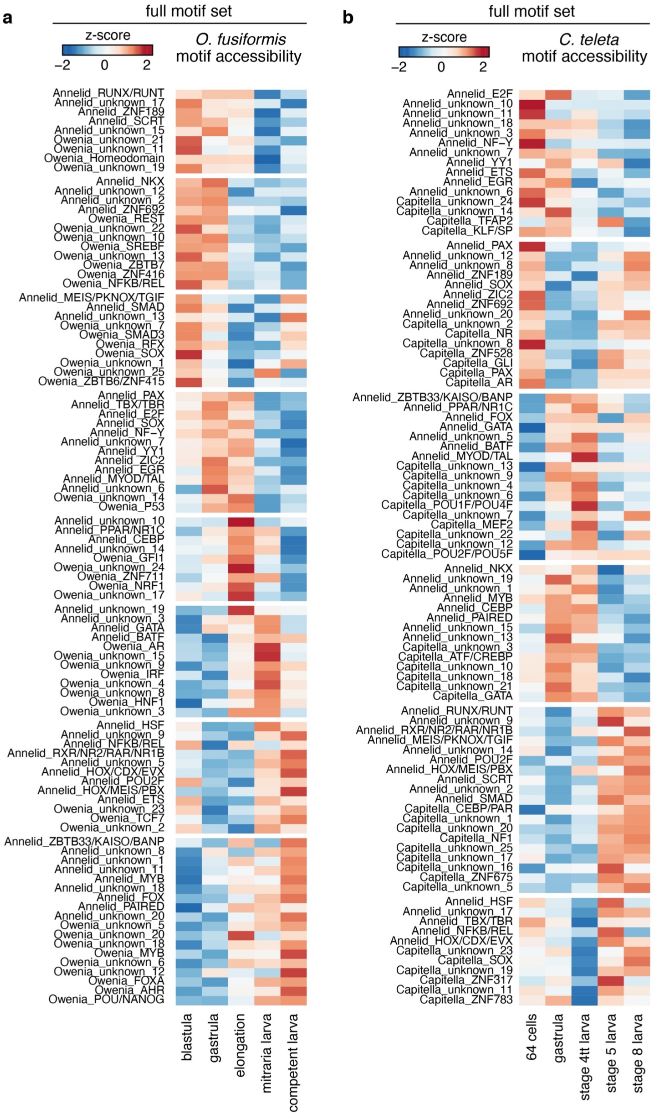

Supplementary Figure 25 | Accessibility dynamics of the full motif archetype set. a, b, Clustered heatmaps of motif archetype accessibility dynamics during *O. fusiformis* (**a**) and *C. teleta* (**b**) development. Custom names depicting putative transcription factors or transcription factor families bound to each motif archetype are shown to the left of the heatmaps. Motifs can be either species-specific (preceded by "Owenia" or "Capitella") or common to both annelids and likely conserved across Annelida (preceded by "Annelid").

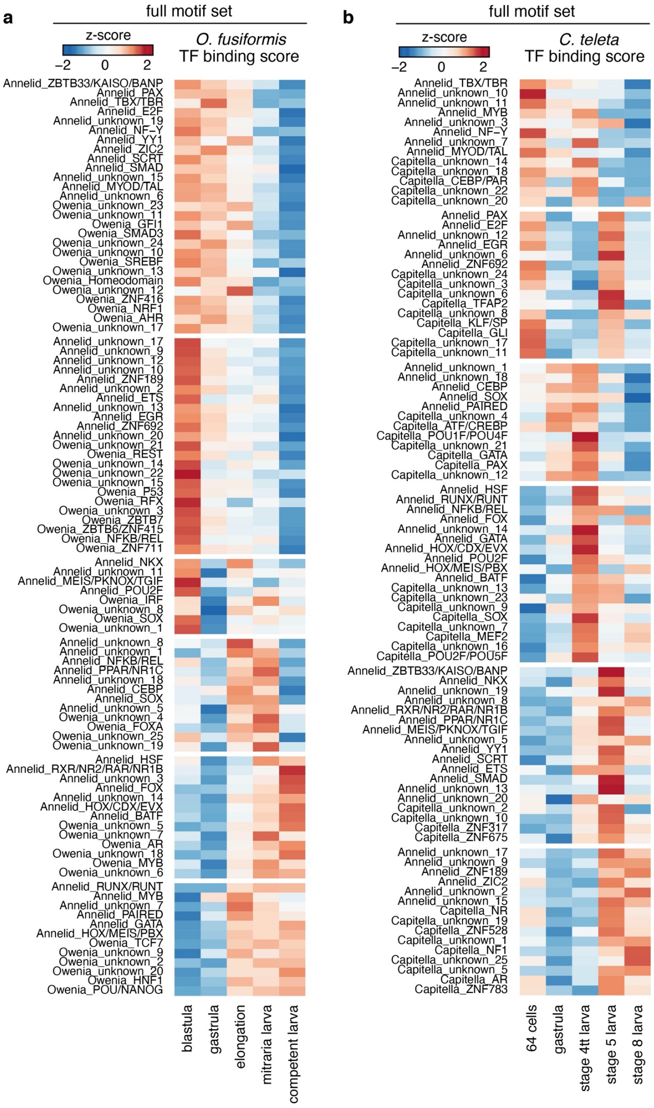

Supplementary Figure 26 | Transcription factor footprinting of the full motif archetype set. a, b, Clustered heatmaps of transcription factor binding score dynamics for each motif archetype inferred from transcription factor footprinting during *O. fusiformis* (**a**) and *C. teleta* (**b**) development. Custom names depicting putative transcription factors or transcription factor families bound to each motif archetype are shown to the left of the heatmaps. Motifs can be either species-specific (preceded by "Owenia" or "Capitella") or common to both annelids and likely conserved across Annelida (preceded by "Annelid").

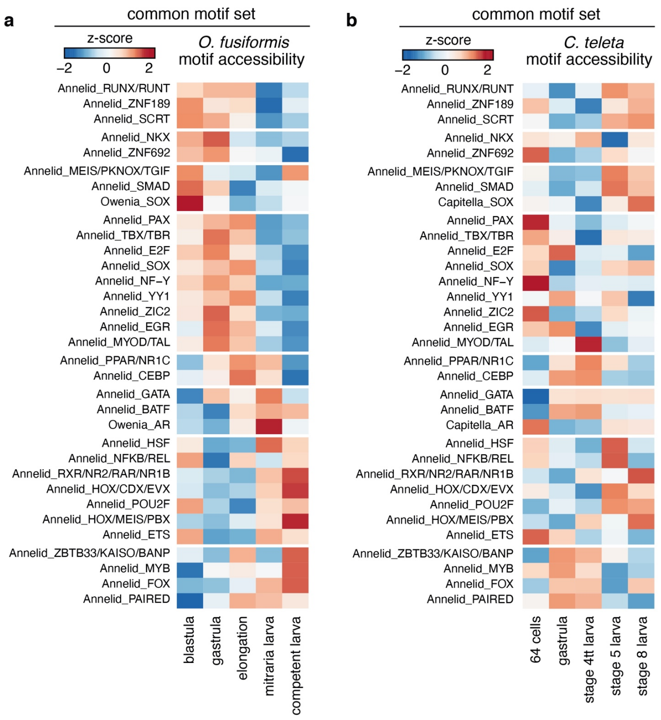

Supplementary Figure 27 | Accessibility dynamics of the common annelid motif archetype set. a, b, Clustered heatmaps of motif archetype accessibility dynamics during *O. fusiformis* (**a**) and *C. teleta* (**b**) development. Only archetypes common to both annelids are shown. Custom names depicting putative transcription factors or transcription factor families bound to each motif archetype are shown to the left of the heatmaps. Depicted species-specific archetypes (preceded by “Owenia” or “Capitella”) are shown when there is an unclustered ortholog archetype in the other species (e.g., Owenia_SOX and Capitella_SOX).

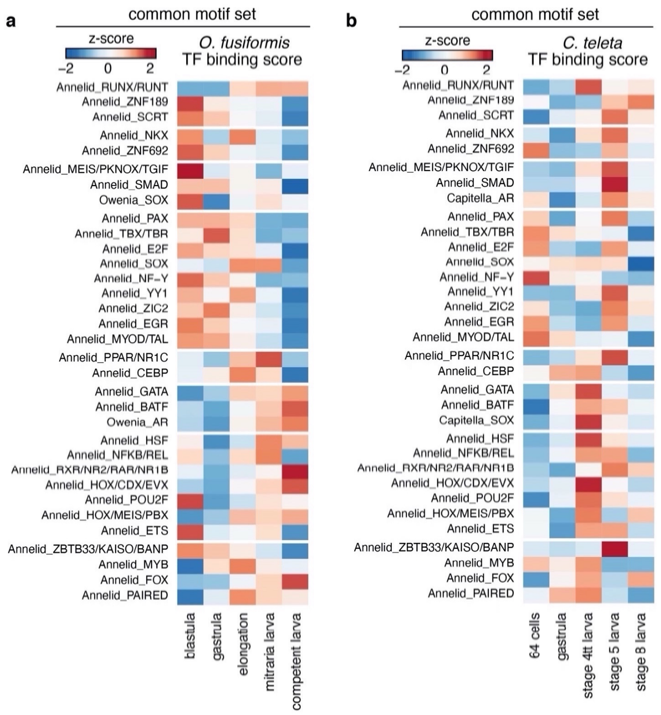

Supplementary Figure 28 | Transcription factor footprinting of the common annelid motif archetype set. a, b, Clustered heatmaps of transcription factor binding score dynamics for each motif archetype inferred from transcription factor footprinting during *O. fusiformis* (**a**) and *C. teleta* (**b**) development. Only archetypes common to both annelids are shown. Custom names depicting putative transcription factors or transcription factor families bound to each motif archetype are shown to the left of the heatmaps. Depicted species-specific archetypes (preceded by "Owenia" or "Capitella") are shown when there is an unclustered ortholog archetype in the other species (e.g., Owenia_SOX and Capitella_SOX).

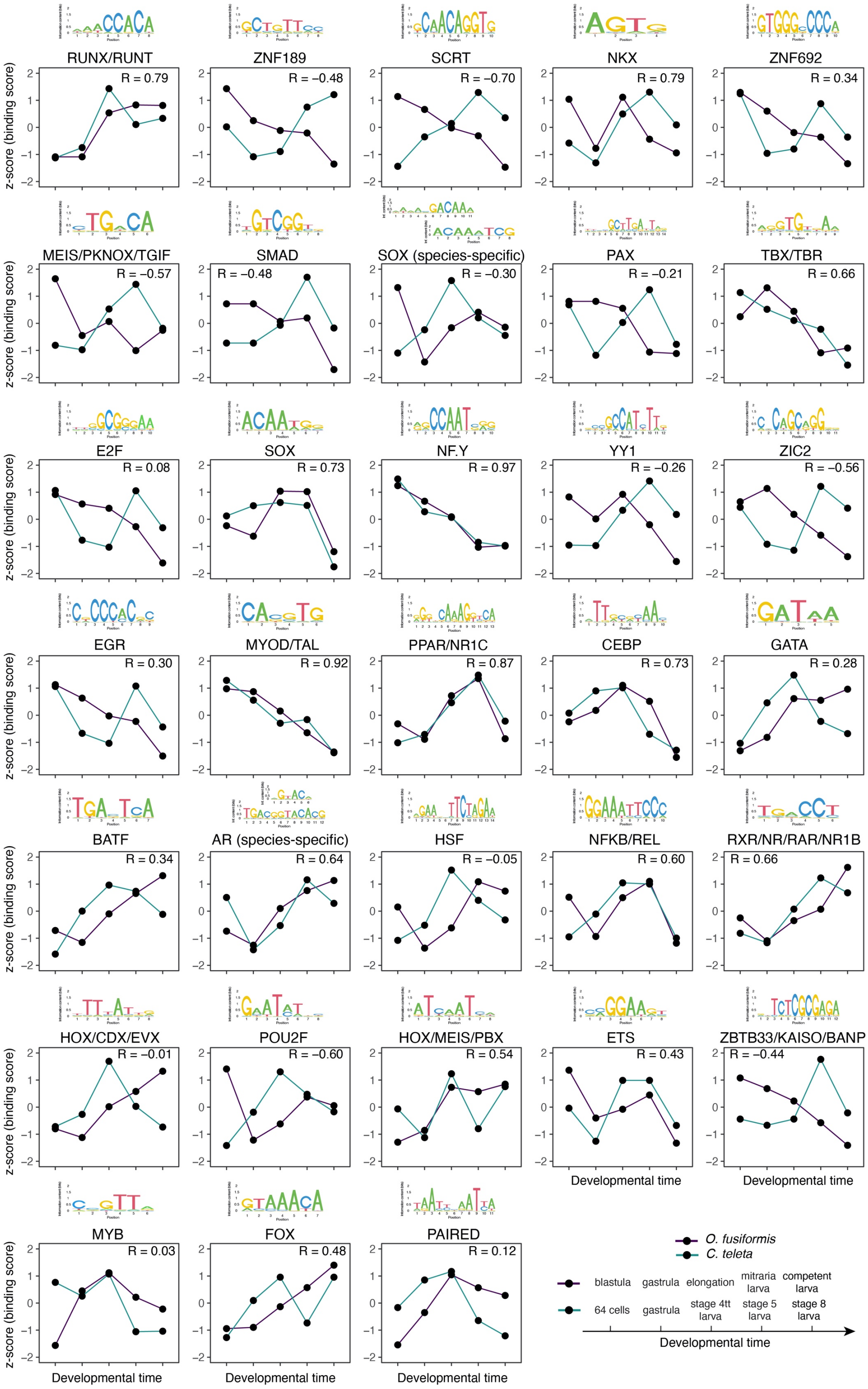

Supplementary Figure 29 | Cross-species comparison of transcription factor binding dynamics in the common annelid motif archetype set. Transcription factor binding score dynamics for each motif archetype inferred from transcription factor footprinting during *O. fusiformis* (purple) and *C. teleta* (blue) development. Only archetypes common to both annelids are shown. Sequence logos are shown on top of each plot. R: Pearson correlation coefficients derived from two-tailed tests.

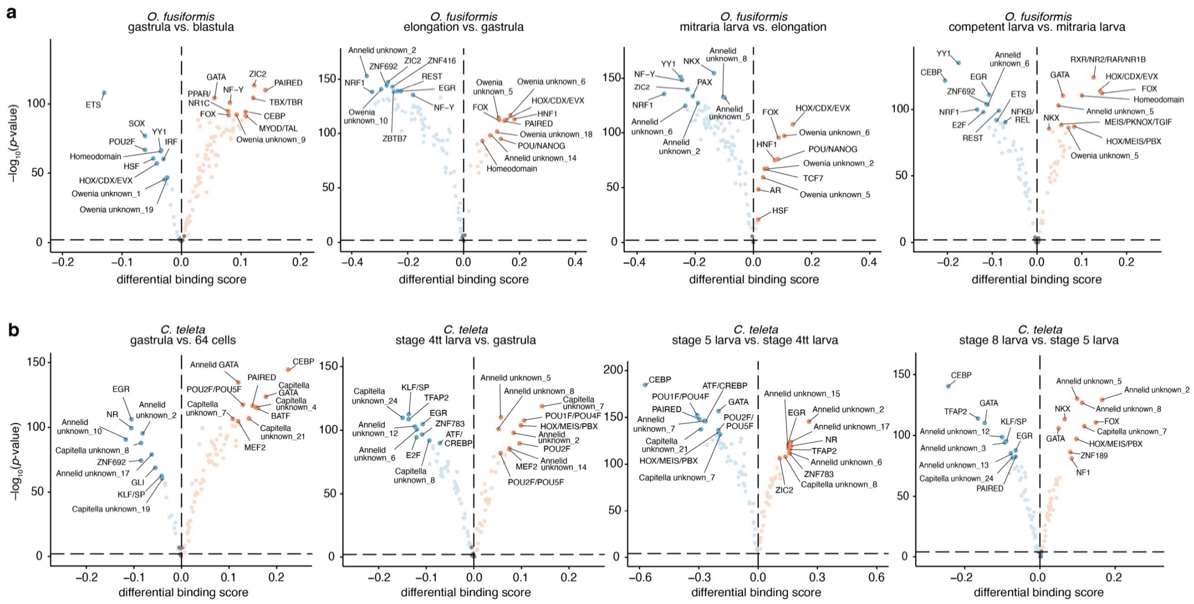

Supplementary Figure 30 | Differential transcription factor binding. a, b, Volcano plots of differential transcription factor binding to each motif archetype from pair-wise comparisons between consecutive developmental stages for *O. fusiformis* (**a**) and *C. teleta* (**b**). Upregulated or more bound archetypes are in red, downregulated or less bound archetypes are in blue. Motif archetype names for top 10 upregulated and downregulated archetypes are highlighted. Where unambiguous, the "Annelid" prefix was dropped from the name. *P*-values were derived using the described TOBIAS pipeline.

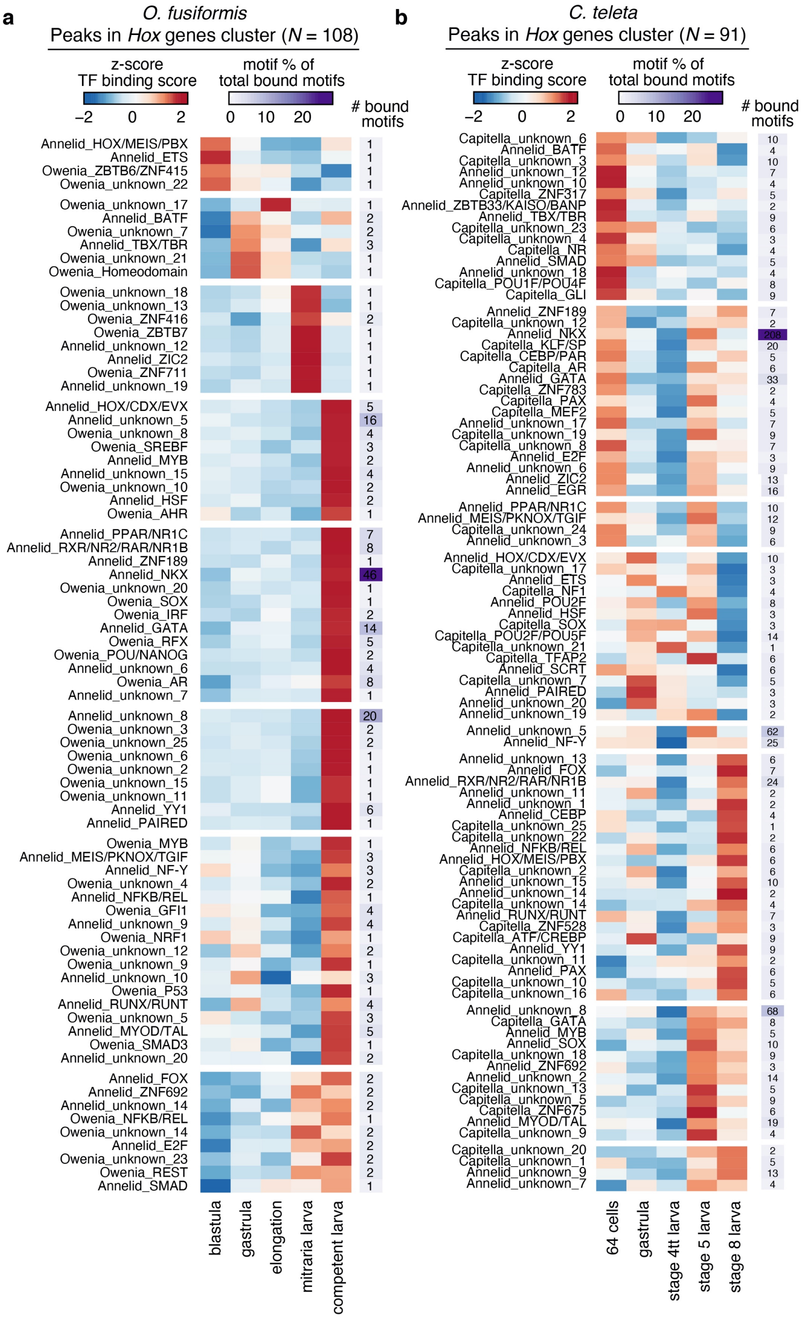

Supplementary Figure 31 | Transcription factor binding dynamics in peaks of the *Hox* genes cluster. a, b, Clustered heatmaps of transcription factor binding score for each motif archetype inferred from transcription factor footprinting during *O. fusiformis* (**a**) and *C. teleta* (**b**) development, restricted to the 108 (*O. fusiformis*) and 91 peaks (*C. teleta*) annotated to a gene of the *Hox* genes cluster (i.e., all *Hox* genes but *Post1*). The proportion of bound archetypes that each motif represents is colour-coded in purple on the right side of each heatmap. Absolute number of bound motif archetypes are inside the purple colour-coded cells. Custom names depicting putative transcription factors or transcription factor families bound to each motif archetype are shown to the left of the heatmaps. Even though both species show developmental dynamics, up to 57 motif archetypes (up to 76 % of the bound total) are strongly preferentially bound at the competent larva of *O. fusiformis*. This suggests a generalised compaction and inaccessibility for transcription factor binding in the *Hox* genes cluster until after the mitraria larva stage, when *Hox* genes become deployed in *O. fusiformis*.

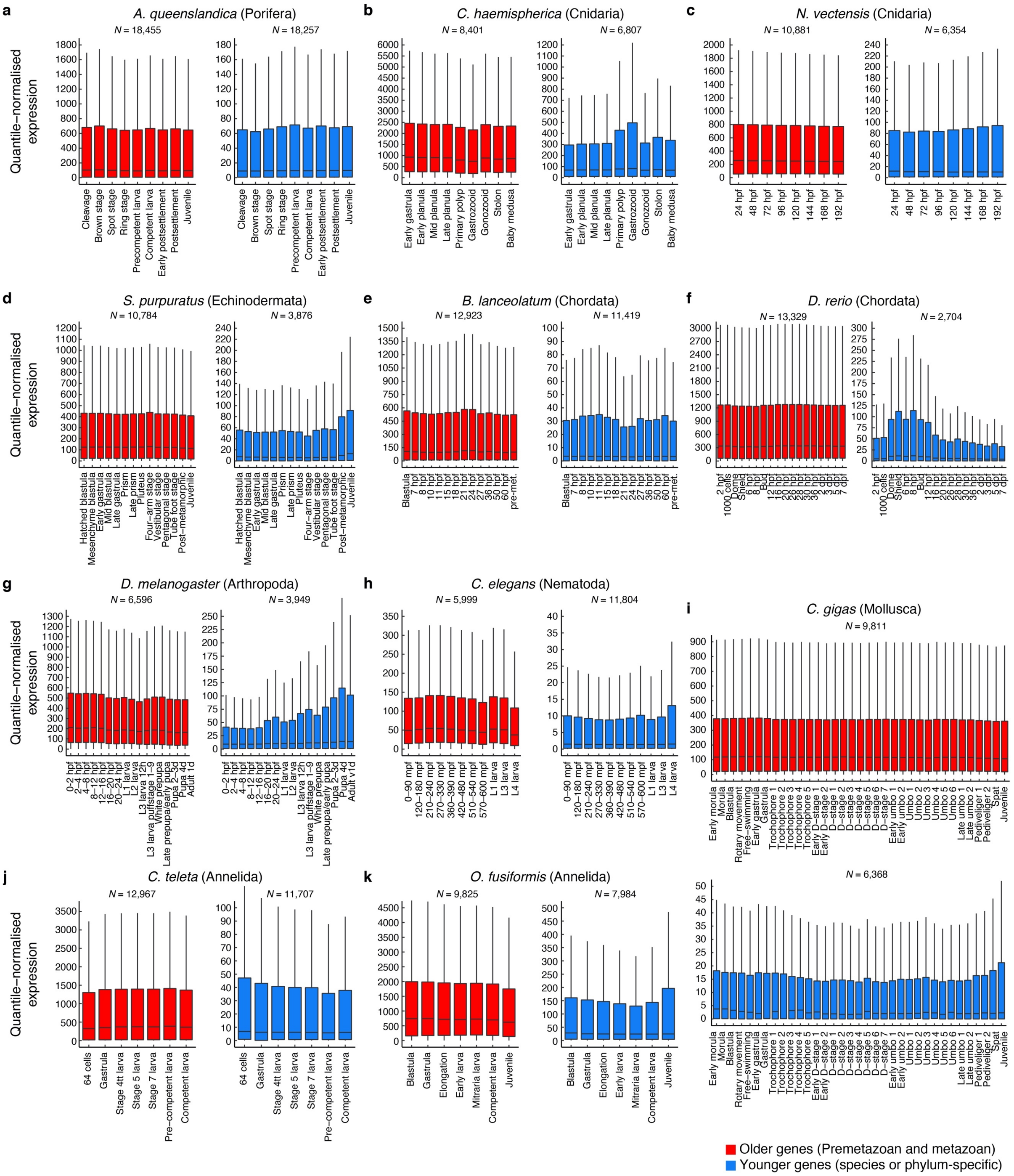

Supplementary Figure 32 | Developmental expression dynamics of phylum- and species-specific genes across Metazoa. a–i Boxplots of quantile-normalised expression levels of younger genes (blue, right, from the phylum- and species-specific phylostratum) opposed to older genes (red, left, from the pre-metazoan and metazoan phylostratum) during development of *A. queenslandica* (**a**), *C. hemisphaerica* (**b**), *N. vectensis* (**c**), *S. purpuratus* (**d**), *B. lanceolatum* (**e**), *D. rerio* (**f**), *D. melanogaster* (**g**), *C. elegans* (**h**), *C. gigas* (**i**), *C. teleta* (**j**), and *O. fusiformis* (**k**). Centre lines, median; box, interquartile range (IQR); whiskers, first or third quartile ± 1.5 × IQR. Lower whiskers are sometimes not apparent due to the distribution skewness towards zero.

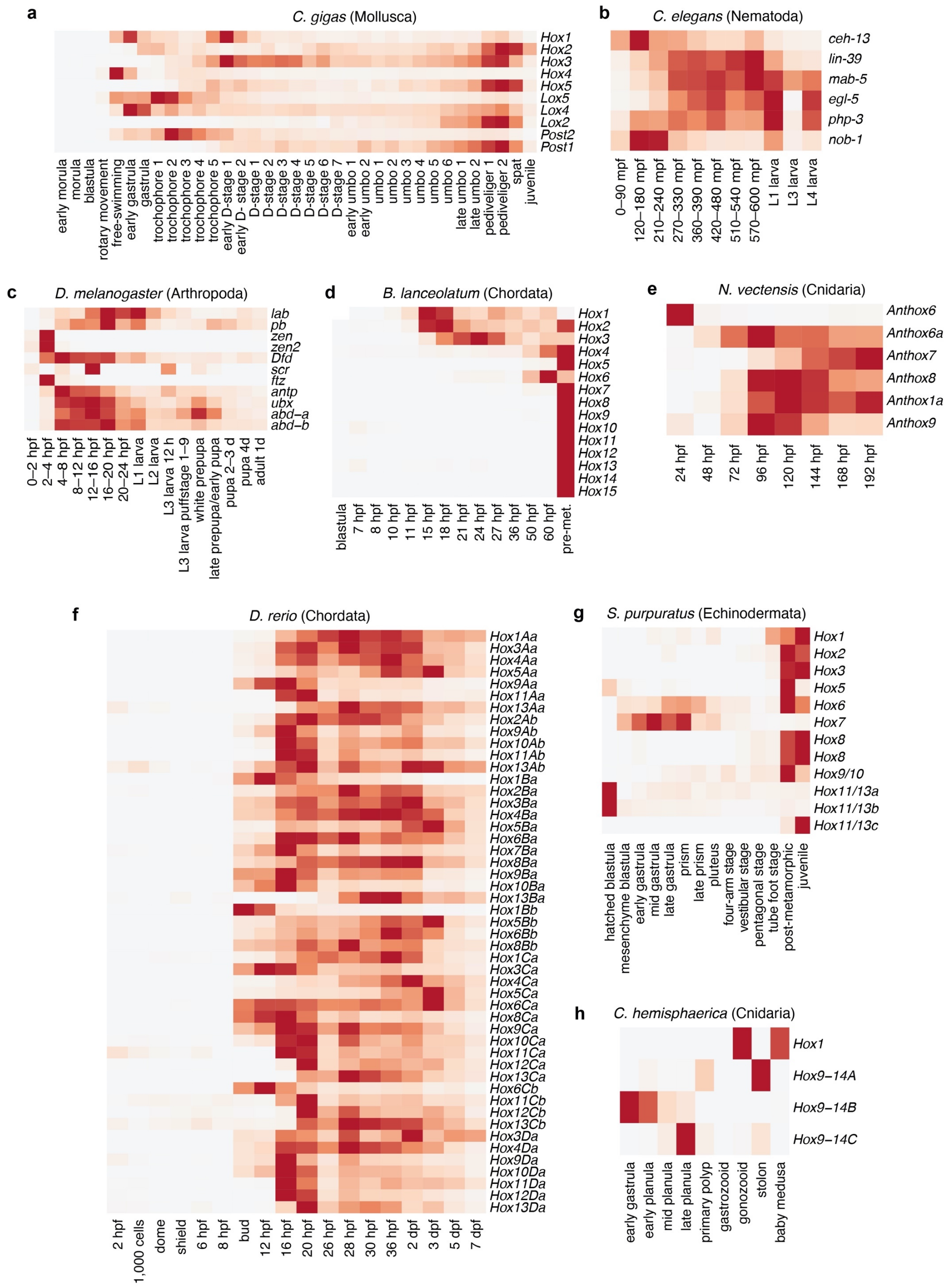

Supplementary Figure 33 | *Hox* genes developmental expression dynamics across Metazoa. a–h Fully labelled heatmaps of *Hox* gene expression during development for *C. gigas* (**a**), *C. elegans* (**b**), *D. melanogaster* (**c**), *B. lanceolatum* (**d**), *N. vectensis* (**e**), *D. rerio* (**f**), *S. purpuratus* (**g**), and *C. hemisphaerica* (**h**). Equivalent heatmaps for *O. fusiformis*, *C. teleta*, and *U. unicinctus* can be found in Extended Data Figure 5d. *A. queenslandica* is not here shown because it lacks *Hox* genes. Gene identifiers for every gene are listed in Supplementary Table 94.
