# Supplementary material for: Annelid functional genomics reveal the origins of bilaterian life cycles
Source: Nature. 2023 Jan 25;615(7950):105–10. doi: 10.1038/s41586-022-05636-7 (PMC9977687; doi:10.1038/s41586-022-05636-7)
Supplement: Supplementary file 2 — Reporting Summary [file 41586_2022_5636_MOESM2_ESM.pdf]

nature portfolio

Corresponding author(s): José M Martín-Durán, Ferdinand Marlétaz

Last updated by author(s): Nov 24, 2022

Reporting Summary

Nature Portfolio wishes to improve the reproducibility of the work that we publish. This form provides structure for consistency and transparency in reporting. For further information on Nature Portfolio policies, see our Editorial Policies and the Editorial Policy Checklist.

Statistics

For all statistical analyses, confirm that the following items are present in the figure legend, table legend, main text, or Methods section.

n/a	Confirmed	
☐	☒	The exact sample size (*n*) for each experimental group/condition, given as a discrete number and unit of measurement
☐	☒	A statement on whether measurements were taken from distinct samples or whether the same sample was measured repeatedly
☐	☒	The statistical test(s) used AND whether they are one- or two-sided<br>*Only common tests should be described solely by name; describe more complex techniques in the Methods section.*
☐	☒	A description of all covariates tested
☐	☒	A description of any assumptions or corrections, such as tests of normality and adjustment for multiple comparisons
☐	☒	A full description of the statistical parameters including central tendency (e.g. means) or other basic estimates (e.g. regression coefficient) AND variation (e.g. standard deviation) or associated estimates of uncertainty (e.g. confidence intervals)
☐	☒	For null hypothesis testing, the test statistic (e.g. *F*, *t*, *r*) with confidence intervals, effect sizes, degrees of freedom and *P* value noted<br>*Give P values as exact values whenever suitable.*
☐	☒	For Bayesian analysis, information on the choice of priors and Markov chain Monte Carlo settings
☒	☐	For hierarchical and complex designs, identification of the appropriate level for tests and full reporting of outcomes
☐	☒	Estimates of effect sizes (e.g. Cohen's *d*, Pearson's *r*), indicating how they were calculated

Our web collection on statistics for biologists contains articles on many of the points above.

Software and code

Policy information about availability of computer code

Data collection	SRA-Toolkit v2.11.3
Data analysis	Jellyfish v.2.3, FloMax v.2.82, GenomeScope 2.0, CANU v.8.3rc2, bwa mem v.0.7.17, Racon v.1.16, purge_haplotigs v.1.0.4, Merqury v.1.1, BUSCO v.5, RepeatModeler v.2.0.1, RepBase, DIAMOND v.0.9.22, RepeatMasker "open-4.0", LTR_finder v.1.07, RepeatCraft v.0.1.1, SAMtools v.1.9, STAR v. 2.5.3a, trimmomatic v.0.39, StringTie v.1.3.6, Portcullis v.1.1.2, Trinity v.2.5.1, GMAP v.2020-04-08, Mikado v.2.1 pipeline, BLAST v.2.2.31+, Augustus v.3.2.3, Exonerate v.2.4.0, Augustus v.3.2.3, PASA v.2.3.3, Trinotate v.3.0, HMMER v.2.3.2, signalP v.4.1, PANTHER HMM, Juicer pipeline r.e0d1bb7, 3d-dna v.180419, Juicebox v.1.11.08, Liftoff v.1.6.1, AGAT suite of scripts v0.8.1, OrthoFinder v.2.2.7, MMSeqs2, ETE 3 library, kallisto v.0.46.2, ESeq2 v.1.30.1 package, mfuzz v.2.52 package, WGCNA package v.1.70–3, Cytoscape v.3.8.2, topGO v.2.44, simplifyEnrichment v.1.2.0 package, MAFFT v.7, IQ-TREE v.2.0.3, MrBayes v.3.2.7a, BlastKOALA server, gBlocks v.0.91b, RAxML v.8.2.11.9, FigTree v.1.4.4, cutadapt v.2.5, NextGenMap v.0.5.5, deepTools v.3.4.3, MACS2 v.2.2.7.1, BEDtools v.2.28.0, IDR v.2.0.4.2, DiffBind v.3.0.14, UpSetR v.1.4.0, HOMER v.4.1, pyGenomeTracks v.2.1, GimmeMotifs v.0.16.1, TOBIAS v.0.12.0, scikit-learn v1.0.2, philentropy v.0.5.0 package, R version 3.5.1, R version 4.1.2, Python 3.8.10, Visual Studio Code v.1.70.2, Inkscape 1.0.1, Adobe Photoshop 2021 22.4.3. release, Adobe Illustrator 2021 25.4.1 release<br><br>All custom code not previously published and key files relevant for the reproducibility of this study are available in our GitHub repository: https://github.com/ChemaMD/OweniaGenome.

For manuscripts utilizing custom algorithms or software that are central to the research but not yet described in published literature, software must be made available to editors and reviewers. We strongly encourage code deposition in a community repository (e.g. GitHub). See the Nature Portfolio guidelines for submitting code & software for further information.

Data

Policy information about availability of data

All manuscripts must include a data availability statement. This statement should provide the following information, where applicable:

- Accession codes, unique identifiers, or web links for publicly available datasets
- A description of any restrictions on data availability
- For clinical datasets or third party data, please ensure that the statement adheres to our policy

Accession codes and unique identifiers to previously publicly available datasets we used for this study are listed in Supplementary Table 2 (genome files used in gene family evolution analyses), Supplementary Table 8 (transcriptomes used in the evolutionary analysis of chordin in annelids), Supplementary Tables 41 and 43 (gene identifiers used in pathway analyses), Supplementary Table 47 (sequence identifiers used in Hox genes orthology assignment), Supplementary Table 48 (RNA-seq datasets used for Hox gene expression profiling in U. unicinctus) and Supplementary Table 88 (RNA-seq datasets used for comparative annelid and metazoan transcriptomics and Hox gene expression profiling). Repetitive elements database RepBase can be accessed at https://www.girinst.org/repbase/. Transcription factor public database TFClass can be found at http://tfclass.bioinf.med.uni-goettingen.de/. All sequence data associated with this project are available at the European Nucleotide Archive (project PRJEB38497) and Gene Expression Omnibus (accession numbers GSE184126, GSE202283, GSE192478, GSE210813 and GSE210814). Genome assemblies, transposable element annotations, genome annotation files used for RNA-seq and ATAC-seq analyses, WGCNA nodes and edges files, alignment files used in orthology assignment, and other additional files are publicly available in https://github.com/ChemaMD/OweniaGenome.

Field-specific reporting

Please select the one below that is the best fit for your research. If you are not sure, read the appropriate sections before making your selection.

☒ Life sciences ☐ Behavioural & social sciences ☐ Ecological, evolutionary & environmental sciences

For a reference copy of the document with all sections, see nature.com/documents/nr-reporting-summary-flat.pdf

Life sciences study design

All studies must disclose on these points even when the disclosure is negative.

Sample size	Sample sizes for genomic and transcriptomic analyses were estimated based on the amount of genomic DNA and total RNA obtained per individual. For ATAC-seq analyses, sample size per library was that such that there was a final number of 50,000 cells for subsequent tagmentation.
Data exclusions	No data was excluded.
Replication	Two biological replicates were collected for RNA-seq and ATAC-seq datasets, which is a commonly accepted standard in the field. A high correlation between biological replicates was observed. Other experimental techniques (e.g. in situ hybridisation, immunohistochemistry) were performed at least three times to verify observed results.
Randomization	All Capitella teleta animal cultures were set up from randomly selected late larval stages from distinct larval broods to ensure genetic variability in subsequent generations. Embryos and larvae for experiments were collected from either spontaneous broods collected during weekly siftings, or from mating dishes specifically set up for embryonic and larval stages collection between a randomly selected male and a randomly selected female. All Owenia fusiformis animal collections were also performed randomly for in vitro fertilisations to ensure genetic variability in the progeny. Unlabelled and unidentified animals were randomly dissected to obtain either oocytes or sperm.
Blinding	All animal collections were allocated blindly to any of the replicates of study. Investigators did not know during the data analysis stage about the origin of each biological replicate for transcriptomic and epigenomic studies.

Reporting for specific materials, systems and methods

We require information from authors about some types of materials, experimental systems and methods used in many studies. Here, indicate whether each material, system or method listed is relevant to your study. If you are not sure if a list item applies to your research, read the appropriate section before selecting a response.

Materials & experimental systems

n/a	Involved in the study	
☐	☒	Antibodies
☒	☐	Eukaryotic cell lines
☒	☐	Palaeontology and archaeology
☐	☒	Animals and other organisms
☒	☐	Human research participants
☒	☐	Clinical data
☒	☐	Dual use research of concern

Methods

n/a	Involved in the study	
☒	☐	ChIP-seq
☐	☒	Flow cytometry
☒	☐	MRI-based neuroimaging

Antibodies

Antibodies used	Mouse anti-acetyl-alpha tubulin Antibody, clone 6-11B-1, 1:800 dilution (Sigma-Aldrich Cat# MABT868, RRID:AB_2819178), Goat anti-Mouse IgG (H+L) Cross-Adsorbed Secondary Antibody, Alexa Fluor 647, 1:800 dilution (Thermo Fisher Scientific Cat# A-21235, RRID:AB_2535804)
Validation	Antibody cross-reactivity against Owenia fusiformis was predicted based on multiple sequence alignments (MSA) of targeted antigens with closely phylogenetically related species (e.g. Capitella teleta, Platynereis dumerilii, Owenia collaris, etc.) for which there was published literature using those antigens. Antibodies were then validated in immunohistochemistry with our own species.

Animals and other organisms

Policy information about studies involving animals; ARRIVE guidelines recommended for reporting animal research

Laboratory animals	Capitella teleta Blake, Grassle & Eckelbarger, 2009: we kept year round individuals from both sexes and all ages. Adult specimens were only kept until they were 18 weeks old. In this study we only studied embryonic and larval stages.
Wild animals	This study did not involve wild animals.
Field-collected samples	Owenia fusiformis Delle Chiaje, 1844: sexually mature individuals were collected from subtidal waters near the Station Biologique de Roscoff, sent through post to our home institution, and cultured in the lab as described before (see Methods). Animals were kept until the end of their lives or until they were used for experiments, i.e. spawnings for in vitro fertilisations.<br>Magelona spp.: were collected in muddy sand from the intertidal of Berwick-upon-Tweed, Northumberland, NE England (~55.766781, -1.984587) and kept initially in aquaria at the National Museum Cardiff before their transfer to Queen Mary University of London, where they were kept in aquaria with artificial sea water. Magelona spp. were killed for spawnings for in vitro fertilisations too, in order to get larvae for immunohistochemistry.
Ethics oversight	Work on annelids and their embryos are not subject of ethical approvals or restrictions in the United Kingdom.

Note that full information on the approval of the study protocol must also be provided in the manuscript.

Flow Cytometry

Plots

Confirm that:

☒ The axis labels state the marker and fluorochrome used (e.g. CD4-FITC).

☒ The axis scales are clearly visible. Include numbers along axes only for bottom left plot of group (a 'group' is an analysis of identical markers).

☒ All plots are contour plots with outliers or pseudocolor plots.

☒ A numerical value for number of cells or percentage (with statistics) is provided.

Methodology

Sample preparation	Owenia adults were removed from their tubes before flow cytometry analysis. Worms were transferred into a petri dish, washed well in seawater to remove any contaminant, and finely chopped with a razor blade in 2 ml of General-Purpose Buffer to generate a suspension of nuclei. This suspension was filtered through a 30 µm nylon mesh and stained with propidium iodide (Sigma; 1 mg/mL) on ice.
Instrument	We used a flow cytometer Partec CyFlow Space fitted with a Cobalt Samba green laser (532nm, 100mW)
Software	We used the built-in instrument software FloMax v.2.82.
Cell population abundance	We used flow cytometry to estimate genome size using D. melanogaster as reference and thus we did not sort any cell populations. To estimate genome size from propidium iodide staining, we did three independent runs for each species analysing at least 1,000 nuclei per run.
Gating strategy	We considered all cell populations for genome size estimation, and thus no gating strategy was implemented.

☒ Tick this box to confirm that a figure exemplifying the gating strategy is provided in the Supplementary Information.
